# Supernova: A Versatile Vector System for Single-Cell Labeling and Gene Function Studies *in vivo*

**DOI:** 10.1038/srep35747

**Published:** 2016-10-24

**Authors:** Wenshu Luo, Hidenobu Mizuno, Ryohei Iwata, Shingo Nakazawa, Kosuke Yasuda, Shigeyoshi Itohara, Takuji Iwasato

**Affiliations:** 1 Division of Neurogenetics, National Institute of Genetics, Mishima, 411-8540, Japan; 2 Department of Genetics, SOKENDAI (The Graduate University for Advanced Studies), Mishima, 411-8540, Japan; 3 Laboratory for Behavioral Genetics, RIKEN Brain Science Institute, Wako, 351-0198, Japan

## Abstract

Here we describe “Supernova” series of vector systems that enable single-cell labeling and labeled cell-specific gene manipulation, when introduced by in utero electroporation (IUE) or adeno-associated virus (AAV)-mediated gene delivery. In Supernova, sparse labeling relies on low TRE leakage. In a small population of cells with over-threshold leakage, initial tTA-independent weak expression is enhanced by tTA/TRE-positive feedback along with a site-specific recombination system (e.g., Cre/loxP, Flpe/FRT). Sparse and bright labeling by Supernova with little background enables the visualization of the morphological details of individual neurons in densely packed brain areas such as the cortex and hippocampus, both during development and in adulthood. Sparseness levels are adjustable. Labeled cell-specific gene knockout was accomplished by introducing Cre/loxP-based Supernova vectors into floxed mice. Furthermore, by combining with RNAi, TALEN, and CRISPR/Cas9 technologies, IUE-based Supernova achieved labeled cell-specific gene knockdown and editing/knockout without requiring genetically altered mice. Thus, Supernova system is highly extensible and widely applicable for single-cell analyses in complex organs, such as the mammalian brain.

The mammalian brain, a complex organ, comprises numerous cells (neurons) densely packed and interconnected with each other to form intricate neural circuits responsible for higher brain function. To understand the precise cellular and molecular mechanisms of the neural circuit development and function, single-cell analyses that dissect connectivity of individual cells and molecular machinery operating in these cells are indispensable. For this purpose, two transgenic/knock-in mouse-based genetic systems, MADM^1^,^2^ and SLICK^3^, have been reported and have received much attention as promising tools^4^,^5^,^6^. However, unfortunately the use of each system was hampered by its intrinsic weakness (See Discussion). Moreover, systems that solely rely on mouse genetics, such as MADM and SLICK, have common weaknesses, including extensive cost and space requirements for mouse breeding and slow experimental turnover time, making these systems inflexible and hampering their application.

Currently, as alternatives to transgenic/knock-in mouse approaches, in utero electroporation (IUE)-based and virus-mediated gene delivery techniques are widely used for cell labeling and gene manipulation *in vivo*^7^,^8^,^9^,^10^,^11^,^12^,^13^,^14^,^15^,^16^. These methods present extremely rapid experimental turnover, and are applicable for virtually all brain areas and neuron types. Thus, IUE and virus-mediated approaches have many advantages over pure mouse genetic approaches. However, because IUE and virus-mediated systems generally label cells too densely and/or manipulate genes in too many cells in transfected brain areas^7^,^9^,^10^,^16^,^17^,^18^, using IUE or virus systems for single-cell analysis (i.e., sparse cell labeling and labeled cell-specific gene manipulation) has been a challenge requiring methodological innovation. For the purpose of single-cell labeling, some IUE and virus-based methods are recently reported^19^,^20^,^21^,^22^,^23^,^24^,^25^. Nevertheless, further innovation is still necessary for more effective and wider applications (See Discussion). Moreover, for the purpose of achieving sparse and bright cell labeling and labeled cell-specific gene manipulation simultaneously, to date, only the initial primitive version of IUE-based “Supernova” system was briefly described^18^, and further innovation is awaited.

Here we improved and expanded the initial Supernova substantially to develop the Supernova series of vector systems, and characterized these systems in detail to enable wider applications. When Supernova vectors are delivered to a specific brain area using IUE, they achieve sparse cell labeling with extremely high fluorescent intensities and labeled cell-specific gene knockout *in vivo*. Background labeling (ratio of darkly labeled cells) is drastically reduced from the original version. By detailed and systematic characterizations of the systems, we have shown following: (1) The sparseness and brightness of Supernova labeling are constant at different developmental stages and in adulthood. (2) Labeling sparseness is adjustable without affecting the brightness. (3) Supernova enables simultaneous multiple gene expression in a cell. (4) Particularly, the incorporation of RNAi^26^,^27^, transcription activator-like effector nuclease (TALEN)^28^,^29^, and CRISPR/Cas9^16^,^30^ technologies into Supernova is a critical leap. These sets of Supernova achieved single-cell gene knockout/knock-down even without requiring a genetically altered mouse. We further developed adeno-associated virus (AAV)-based Supernova system and demonstrated that it also enables both bright and sparse cell labeling and labeled-cell-specific gene knockout. Thus, Supernova, a promising tool, is simple, rapid, and widely applicable for single-cell analyses of complex organs, including the mammalian brain.

## Results

### The Supernova series of vector systems

**Figure 1:**
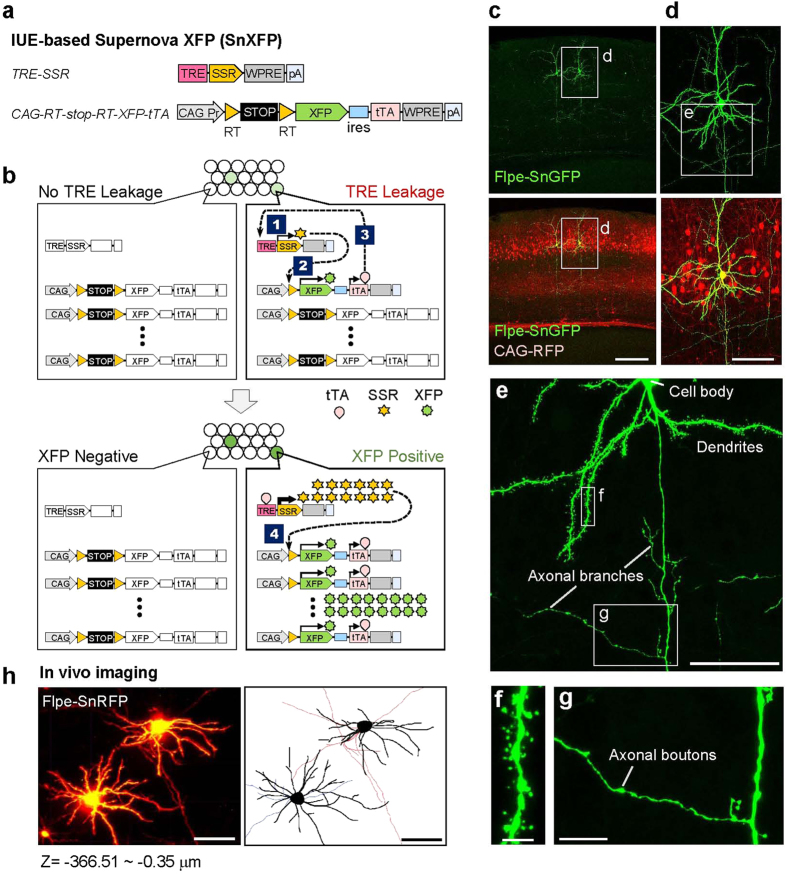
IUE-based Supernova enables sparse and bright cell labeling with little background. (**a**) Schematics showing the elementary components of IUE-based Supernova: TRE-SSR-WPRE-pA (TRE-SSR) and CAG-RT-stop-RT-XFP-ires-tTA-WPRE-pA (CAG-RT-stop-RT-XFP-tTA). TRE: tetracycline response element; tTA: tetracycline transactivator; SSR: site specific recombinase (e.g. Cre, Flpe); RT: recombination target site (e.g. loxP, FRT); XFP: fluorescent proteins (e.g. GFP, RFP); WPRE: WHP post-trascriptional response element; ires: internal ribosome entry site. (**b**) Schematics showing how Supernova works: (1) Initially, only in a sparse population among many cells that are transfected with both vectors, the leakage of TRE drives above-threshold but weak SSR expression. (2) This low level of SSR excises the RT-stop-RT cassette in a few copies of CAG-RT-stop-RT-XFP-tTA vector, initiating the transcription of XFP and tTA, albeit weakly. (3) Through binding with TRE, tTA facilitates expression of SSR. (4) Then RT-stop-RT cassette is excised from many copies of CAG-RT-stop-RT-XFP-tTA vector, and expression of XFP and tTA is increased. This positive loop of tTA/TRE enhancement (See Supplementary Fig. 3) leads to extremely high levels of expression of both SSR and XFP, only in a small population of transfected cells. (**c–g**) Supernova labeling is sparse and bright enough to visualize the detailed morphology of single neurons including dendritic spines (**f**) and axonal branches and boutons (**g**). An Flpe-based Supernova GFP (Flpe-SnGFP) vector set (TRE-Flpe and CAG-FRT-stop-FRT-GFP-tTA) was introduced into layer 2/3 (L2/3) cortical neurons by in utero electroporation (IUE). The CAG-RFP vector was co-electroporated to mark the transfected neurons. Higher-power images of the rectangles in **c** were shown in (**d**). The square in (**d)** were further magnified in (**e**). (**f,g**) Show higher-magnification images of the rectangles in (**e**). (**h**) Two-photon *in vivo* imaging of L4 cortical neurons labeled by Flpe-based Supernova RFP (Flpe-SnRFP) in P5 mouse. The traces of imaged cortical neurons were shown in right panel. Black lines indicate the dendrites of labeled neurons. The axons of these neurons are represented by red and blue lines, separately. Scale bars, 250 μm (**c**); 100 μm (**d**); 50 μm (**e,h**); 4 μm (**f**); 10 μm (**g**).

To allow sparse and bright cell labeling and labeled cell-specific gene manipulation in the mammalian brain, we developed Supernova series of vector systems that use IUE for vector delivery. The elementary composition of IUE-based Supernova includes a set of two vectors: TRE-SSR-WPRE-pA [TRE-SSR; TRE: tetracycline response element; SSR: site-specific recombinase, such as Cre, Flpe^31^ and Dre^32^] and CAG-RT-stop-RT-XFP-ires-tTA-WPRE-pA (CAG-RT-stop-RT-XFP-tTA; RT: recombination target site, such as loxP, FRT, and rox; XFP: fluorescent proteins, such as GFP and RFP; tTA: tetracycline transactivator) (Fig. 1a). The initial Supernova is composed of TRE-Cre and CAG-loxP-stop-loxP-RFP-tTA vectors^18^. For wider applications, we re-designed the CAG-RT-stop-RT-XFP-tTA vector backbone such that one XFP type can be easily replaced with another XFP type or with any gene of interest (Supplementary Fig. 1 and Supplementary Table 1).

When cells are transfected with a Supernova vector set, in a very small population among these cells, TRE leakage drives above-threshold but weak SSR expression, followed by tTA weak expressions. Then, only in these sparse cells, tTA binds with TRE, which further facilitates XFP expression through positive feedback cycles (Fig. 1b). IUE was employed to transfect Supernova vectors into cells in objective brain regions, including each cortical layer and the hippocampus (Supplementary Fig. 2). The significance of tTA/TRE enhancement in the system was clearly demonstrated (See Supplementary Fig. 3 and its legend).

### IUE-based Supernova enables single-cell labeling with high fluorescence intensity *in vivo*

To enable wider applications of the Supernova system, we systematically and comprehensively evaluated Supernova labeling properties. To estimate sparseness and brightness extents of Supernova labeling, we delivered the Supernova GFP vector set (SnGFP) based on Flpe/FRT^31^ [Flpe-SnGFP: TRE-Flpe and CAG-FRT-stop-FRT-GFP-tTA (CAG-FSF-GFP-tTA)] into L2/3 cortical neurons via IUE at embryonic day 15.5 (E15.5). The CAG-RFP vector was co-electroporated to label the transfected neurons. In brains at postnatal day 22 (P22), a small and sparse population (1.2% ± 0.2%, n = 5 mice) of transfected neurons was GFP-positive (Fig. 1c). Most SnGFP-labeled cells were so bright that morphological details, including dendritic spines and axonal boutons, were clearly visible (Fig. 1d–g). By combining an optical clearing method^33^ and two-photon microscopy, we could image and reconstruct a single L2/3 callosal projection neuron having a long axon, in an intact fixed brain (Supplementary Fig. 4). Furthermore, by introducing Flpe-SnRFP into cortical L4 neurons using IUE at E14.5 and observing at P5 using two-photon microscopy, we could visualize the morphological details of dendrites and axons even *in vivo* (Fig. 1h). Note that *in vivo* imaging of single neurons located in deep cortical layers, such as L4, requires excellent sparseness and brightness. These results indicate that Supernova labeling (Flpe-based version) is extremely sparse and bright.

Next, we evaluated the background level of Supernova labeling by delivering Flpe-SnRFP into L2/3 cortical neurons using IUE at E15.5. Notably, almost all (26/28 cells, four mice) Flpe-SnRFP-labeled cells were so bright that visualizing the whole dendritic morphologies of these cells was possible at P6. Only a few (2/28 cells) RFP-positive cells were defined as dark cells, which failed to label some of the basal dendrites to their tips. Thus, Flpe-Supernova achieved high intensity fluorescent neuronal labeling with little background.

### IUE-based Supernova is applicable for several developmental stages and in adulthood

**Figure 2:**
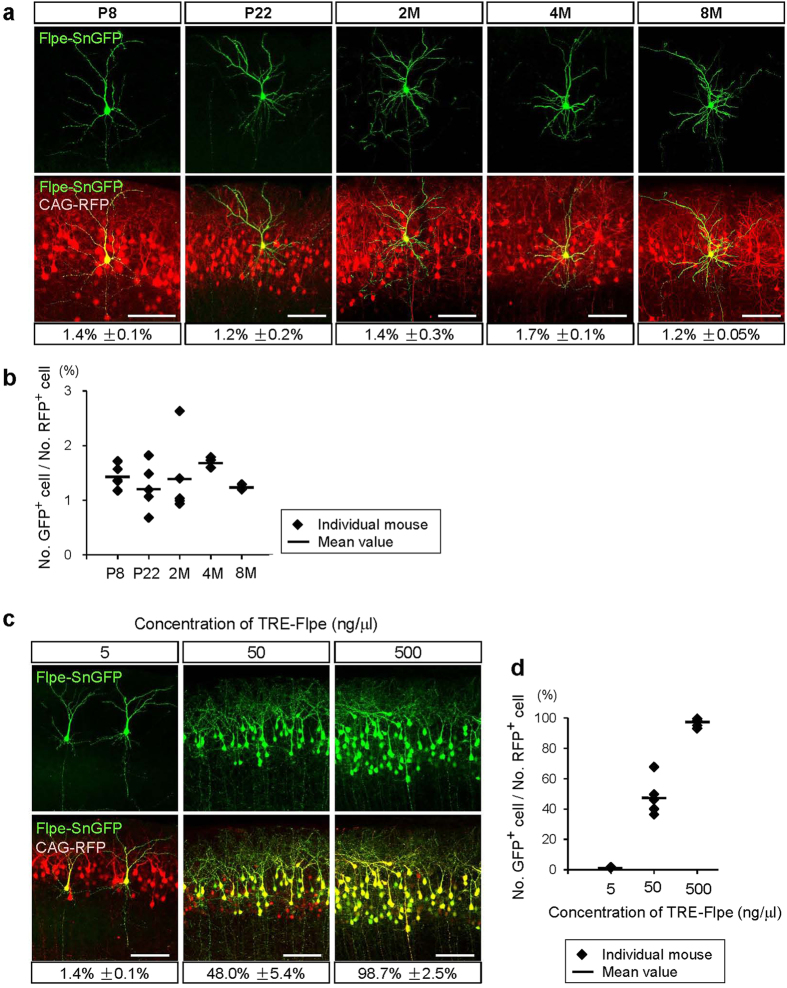
The sparseness of Supernova labeling is stable and adjustable. (**a,b**) The sparseness and brightness of Supernova labeling were constant from early postnatal stages to adulthood. Images of L2/3 cortical neurons labeled by Flpe-SnGFP, in which concentration of the TRE-Flpe vector was 5 ng/μl (standard concentration), were shown (**a**). CAG-RFP was co-electroporated to label all the transfected cells. Coronal sections were made from P8, P22, 2 month-old (2M), 4M and 8M brains. The ratio of Flpe-SnGFP-labeled cell number to transfected cell number at each age was shown under the image in (**a**) and as diagram in (**b**) [mean ± SEM; n = 5 mice at P8 (30/2162 cells), P22 (8/654 cells), 2M (8/562 cells), n = 3 mice at 4M (9/529 cells), n = 2 mice at 8M (7/555 cells)]. Scale bars, 100 μm. (**c,d**) The degree of sparseness of Supernova-labeling is adjustable. Concentrations of TRE-Flpe vector used for IUE were 5 ng/μl, 50 ng/μl and 500 ng/μl [n = 5 mice for each group; 30/2162 cells (5 ng/μl), 890/1880 cells (50 ng/μl), 2507/2539 cells (500 ng/μl)]. L2/3 cortical neurons were labeled by Flpe-SnGFP together with CAG-RFP. Data obtained using 5 ng/μl are common in panels (**a**,**c**). Scale bars, 100 μm.

We quantitatively examined the sparseness of Supernova labeling at different developmental stages and in adulthood by transfecting Flpe-SnGFP and CAG-RFP (control) together. We dissected the brains at P8, P22, 2 months (2 M), 4 M and 8 M (Fig. 2a) and evaluated sparseness as the ratio of Flpe-SnGFP-positive to RFP-positive neurons. The ratios (Fig. 2b) and brightness (Fig. 2a) were similar at all ages examined. Our results imply that the sparseness and brightness of Supernova labeling are constant at different developmental stages and in adulthood.

### Labeling sparseness is adjustable in Supernova system without affecting labeling brightness

We speculate that Supernova-labeling sparseness could be achieved by copy number variability of the TRE-SSR vector among transfected cells. Only cells in which TRE-SSR vector copy numbers are higher than a certain level might drive initial above-threshold SSR expression and finally achieve extremely bright cell labeling by tTA/TRE positive feedback (Fig. 1b). If this scenario is the case, it raises the possibility of adjusting labeling sparseness by changing the TRE-SSR vector concentration in the Supernova vector mixture.

To test this attractive possibility, we prepared a series of Flpe-SnGFP vector mixture, containing different concentrations of the TRE-Flpe vector (5, 50, and 500 ng/μl), and introduced each mixture into L2/3 cortical neurons using IUE at E15.5 (Fig. 2c). The CAG-RFP was co-electroporated to label transfected neurons. The ratio of GFP/RFP-positive cell numbers was quantified at P8 (Fig. 2d). We found that, when 5 ng/μl (standard concentration) was used, only a very small population of RFP-positive neurons (1.4% ± 0.1%, n = 5 mice) was labeled by SnGFP. When 50 ng/μl was used, approximately half of the RFP-positive neurons (48.0% ± 5.4%, n = 5 mice) were SnGFP-positive. When the TRE-Flpe vector concentration was increased to 500 ng/μl, almost all RFP-positive neurons (98.7% ± 2.5%, n = 5 mice) were SnGFP-positive. These data indicate that the sparseness of Supernova labeling is adjustable by simply changing the TRE-SSR vector concentration in the DNA solution for IUE. It is also important that labeling brightness was not altered by changing labeling sparseness.

The initial version of Supernova, which is Cre-based SnRFP, is developed to meet a requirement for *in vivo* imaging of single L4 neurons in the neonatal mouse cortex, and therefore characterization of the system is restricted to the specific research purpose^18^. In the present study, we characterized Cre-based SnXFPs in various brain areas and ages and showed that they achieve sparse and bright cell labeling (Supplementary Figs 1b,c, 2a and 5) similar to that achieved by Flpe-based Supernova. However, we also noticed a few differences between Flpe-based and Cre-based versions (See Supplementary Fig. 6 and the legend for details). First, compared with the fluorescence signals expressed by Flpe-based Supernova, those expressed by Cre-based Supernova became visible more quickly after IUE (Supplementary Fig. 6a–h). Second, when the same TRE-SSR vector concentration was used, the sparseness of Flpe-Supernova labeling was superior to that of Cre-Supernova labeling (Supplementary Fig. 6i). Third, although the background level of Cre-Supernova labeling was low, that of Flpe-Supernova labeling was much lower (superior) (Supplementary Fig. 6c,d,g,h).

In conclusion, although Flpe- and Cre-based versions show some differences, both Supernova versions are useful for visualizing the morphological details of individual neurons in densely packed brain areas, such as the cortex, by achieving sparse and bright cell labeling with low background.

### Supernova enables Cre-independent single-cell labeling in Cre-expressing transgenic mice

To date, many Cre-expressing mouse lines have been reported and have contributed in advancing our understanding of the brain by generating region- and cell type-specific knockout mice^34^,^35^,^36^,^37^. To examine whether Supernova can be used in Cre-expressing mice, we introduced Flpe-SnGFP into the cortical neurons of an Emx1-Cre knock-in mouse, which shows Cre-mediated recombination in all excitatory neurons of the cortex^34^,^38^,^39^. As a recombination indicator, CAG-loxP-stop-loxP-RFP-WPRE (CAG-LSL-RFP) vector was co-expressed (Supplementary Fig. 7a). CAG-LSL-RFP labeling was overcrowded in the cortex, confirming ubiquitous Cre expression in the cortical excitatory neurons of Emx1-Cre mice. In contrast, Flpe-SnGFP labeled only a very small neuron population, even in Cre-expressing cortex; therefore, the morphological details of labeled neurons could be clearly observed (Supplementary Fig. 7b). We also developed Dre/rox-based Supernova, which gave similar results (Supplementary Fig. 8). Thus, Supernova labeling (Flpe- and Dre-versions) is suitable for Cre-independent sparse labeling in Cre-expressing brain areas.

### Supernova enables simultaneous multiple gene expression in a cell

**Figure 3:**
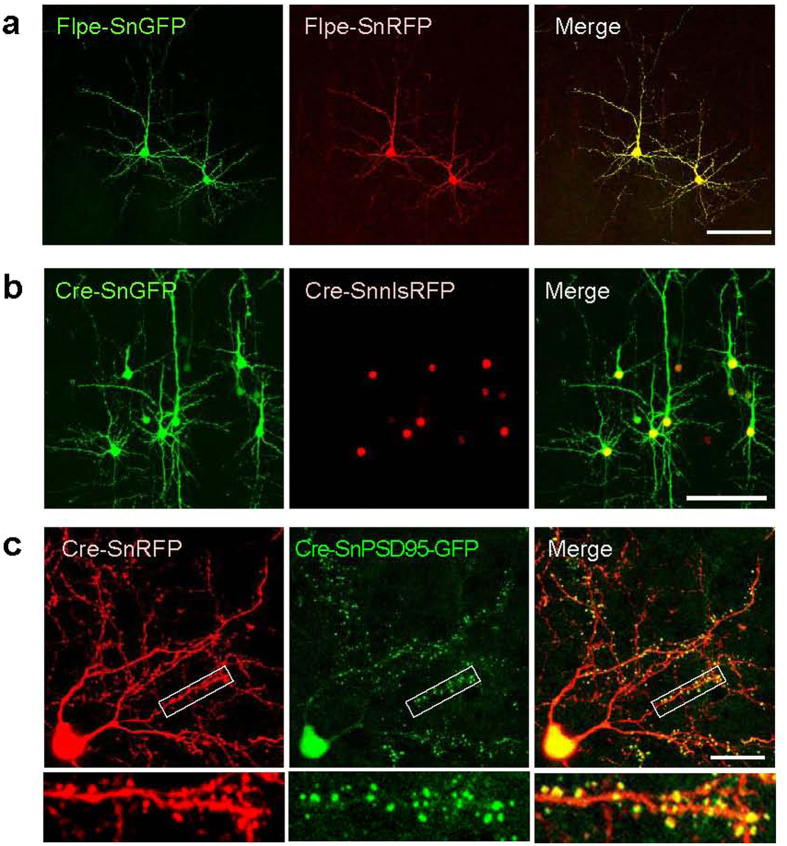
Simultaneous visualization of multiple proteins in a single cell using the Supernova. (**a**) Using the Supernova, RFP and GFP were expressed in sparsely labeled neurons with high co-expression efficiency (RFP^+^/GFP^+^ = 51/53 cells, GFP^+^/RFP^+^ = 51/51 cells, n = 5 mice). Flpe-based Supernova vector sets (TRE-Flpe, CAG-FSF-RFP-tTA and CAG-FSF-GFP-tTA) were introduced by IUE. (**b**) RFP fused with nuclear localization signal (nlsRFP) and GFP was co-expressed in a small population of cortical neurons by introducing Supernova vectors (TRE-Cre, CAG-LSL-GFP-tTA and CAG-LSL-nlsRFP-tTA). (**c**) Simultaneous visualization of RFP and PSD95-GFP in the same single neuron. Upper panel shows low-power images of a tangential section through the somatosensory cortex L4 transfected with SnRFP and Supernova PSD95-GFP (SnPSD95-GFP) vectors (TRE-Cre, CAG-LSL-RFP-tTA and CAG-LSL-PSD95-GFP-tTA). Bottom shows higher-magnification images of the rectangles in the upper panel. Scale bars, 100 μm (**a**,**b**); 25 μm (**c**).

To examine the possibility of simultaneous multiple gene expressions in a sparse cell population, we introduced SnGFP and SnRFP together (i.e., TRE-Flpe, CAG-FSF-GFP-tTA, and CAG-FSF-RFP-tTA) into cortical neurons using IUE at E15.5. We found that at P8, most labeled neurons expressed both GFP and RFP (RFP^+^/GFP^+^ = 51/53 cells, GFP^+^/RFP^+^ = 51/51 cells, n = 5 mice) (Fig. 3a).

Using the simultaneous multiple gene expressions in single cells, we visualized the cell and nucleus together through the co-transfection of SnGFP and Supernova nuclear localization signal (nls)-RFP (SnnlsRFP) into cortical neurons (Fig. 3b). We also simultaneously transfected SnRFP and Supernova postsynaptic density protein (PSD) 95-fused GFP (SnPSD95-GFP) into L4 cortical neurons using IUE, and visualized both whole dendritic morphology and individual spines in single cells with RFP and GFP, respectively (Fig. 3c).

### Supernova system enables sparsely labeled cell-specific gene knockout in floxed mice

**Figure 4:**
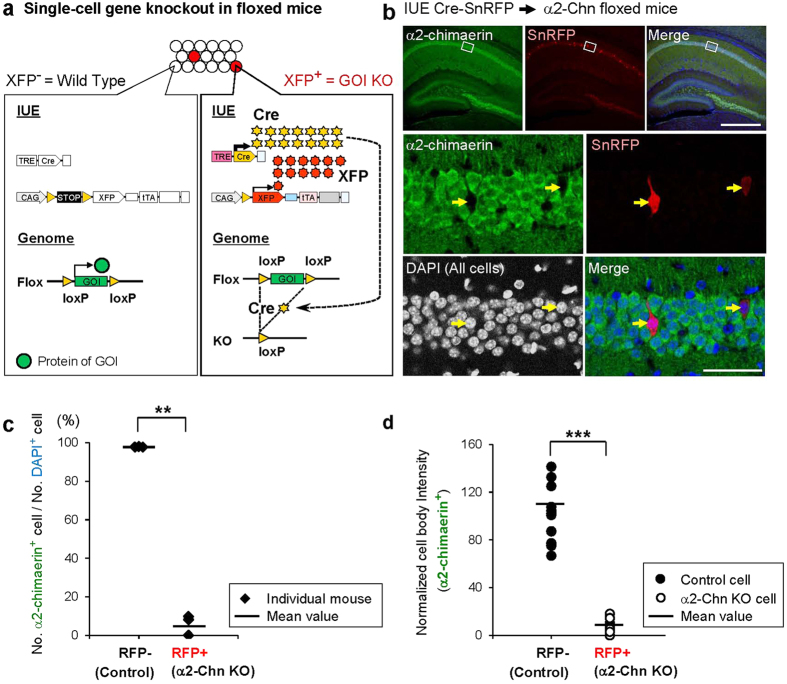
Labeled cell-specific gene knockout via Cre-based Supernova in floxed mice. (**a**) Schematic for Supernova-mediated single-cell knockout (KO) of endogenous gene of interest (GOI) flanked by two loxP sites. (**b**) *α*2-Chimaerin protein is expressed ubiquitously in the hippocampal CA1, while it is undetected specifically in SnRFP-labeled neurons (arrows), indicating that Cre-based Supernova-mediated gene knockout is highly specific to the labeled cells. Cre-SnRFP vectors were introduced into *α2-chimaerin* (*α2-Chn*)^flox/flox^ mouse CA1 by IUE. *α*2-chimaerin immunohistochemistry and DAPI-staining were performed. Upper panels show the hippocampus of *α2-Chn*^flox/flox^ mouse. A set of enlarged example images is shown in the bottom. Note that because extremely high intensity signal of Supernova labeling hinders detection of *α*2-chimaerin signal, we partially photobleached SnRFP signal in this experiment. (**c,d**) Quantification of Supernova-dependent *α2-Chn* knockout efficiency and specificity. (**c**) *α*2-Chimaerin expression was detected in almost all of SnRFP-negative CA1 hippocampal cells (97.7% ± 0.1%, n = 3 mice; 785 cells/804 cells, 464 cells/474 cells, 1011 cells/1036 cells), while only in 5.9% ± 3.0% (n = 3 mice; 3 cells/31 cells, 0 cell/18 cells, 3 cells/37 cells) of SnRFP-positive CA1 neurons, indicating labeled cell-specific knockout. All values represent as mean ± SEM; (**): 0.001 < P < 0.01; Welch’s *t*-test. (**d**) Intensities of *α*2-chimaerin signal in RFP^+^ cells (*α2-Chn* KO cells) and RFP^-^ cells (control cells) that surround RFP^+^ cells were measured (n = 14 cells, 3 mice for each group). (***) P < 0.001, Welch’s *t*-test. Scale bars: 500 μm (**b**, upper); 100 μm (**b**, bottom).

Cre-based Supernova system design predicts high Cre expression levels in Supernova-labeled cells, whereas Cre expression absence in non-labeled cells (Fig. 1b); thus, a floxed gene in the genome is deleted only in Supernova-labeled cells (Fig. 4a).

Here, we systematically and quantitatively analyzed the efficiency and specificities of Cre-based Supernova for knocking out an endogenous floxed gene in the mouse. We chose *α2-chimaerin* (*α2-Chn*) as a target gene because *α*2-chimaerin is ubiquitously expressed in the hippocampal CA1 pyramidal neurons at a high level^40^,^41^ and an anti-*α*2-chimaerin antibody that is applicable for immunohistochemistry is available^42^. We introduced Cre-SnRFP into the hippocampal neurons of *α2-Chn*^flox/flox^ mice using IUE at E14.5 (Fig. 4b). The brains were dissected at P14, when the *α*2-chimaerin signals are strongest in the CA1 region^40^,^41^. *α*2-Chimaerin is ubiquitously expressed in CA1, but specifically lacks in SnRFP-labeled neurons (Fig. 4b). We quantified the ratios of *α*2-chimaerin-positive cells in RFP-negative (control) and RFP-positive (SnRFP-labeled) cells. In the hippocampal CA1 region, 97.7% ± 0.1% of RFP-negative cells expressed *α*2-chimaerin, whereas only 5.9% ± 3.0% of RFP-positive neurons showed *α*2-chimaerin signals (n = 3 mice, Fig. 4c). Furthermore, even in these SnRFP-labeled *α*2-chimaerin-positive neurons (n = 14 cells, three mice), *α*2-chimaerin expression levels were much lower than those in RFP-negative control cells (n = 14 cells, three mice) (Fig. 4d). These results demonstrate extremely high specificity and efficiency of the Supernova-induced gene knockout, and provide compelling evidence that Supernova is suitable for conditional gene knockout using a floxed mouse.

### Supernova-mediated RNAi enables sparsely labeled cell-specific gene knockdown without using genetically altered mice

**Figure 5:**
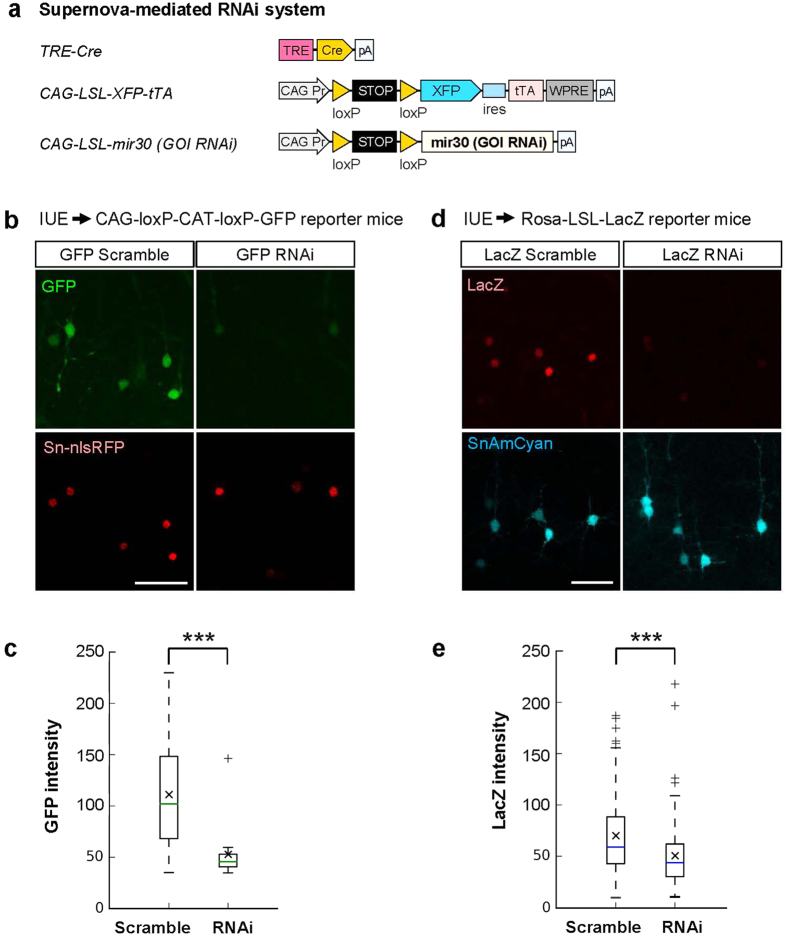
Supernova-mediated RNAi achieved sparsely labeled cell-specific gene knockdown *in vivo*. (**a**) Schematic representation of the Supernova vector set that is for single-cell knockdown of gene of interest (GOI). shRNA against GOI was expressed by CAG-LSL-mir30, which is a Cre-dependent shRNA expression vector^14^. (**b**) CAG-LSL-mir30:GFP-RNAi-Scramble (control: left panels) or CAG-LSL-mir30:GFP RNAi (right panels) was co-electroporated with Supernova nuclear localization signal RFP (Sn-nlsRFP) into CAG-loxP-CAT-loxP-GFP reporter mouse^71^ cortex by IUE at E14.5. Sections were stained with an anti-GFP antibody. Scale bar, 50 μm. (**c**) GFP signals in GFP-RNAi-expressing neurons were significantly lower than those in cells expressing GFP-RNAi-Scramble-expressing neurons (control). Box-and-Whisker plot: the bottom and top of the box are the first and third quartiles; the horizontal line inside the box represents the median; the mean is plotted as a cross. The ends of the whiskers represent the lowest datum within 1.5 interquartile range (IQR) of the lower quartile, and the highest datum within 1.5 IQR of the upper quartile. Data not included between the whiskers were plotted as pluses. (***) < P < 0.001, Brunner-Munzel test. (**d**) CAG-LSL-mir30:LacZ-RNAi-Scramble (control: left panels) or CAG-LSL-mir30:LacZ-RNAi (right panels) was co-transfected with SnAmCyan into the Rosa-loxP-stop-loxP-nlsLacZ (RNZ) reporter mouse^47^ cortex by IUE at E14.5. Coronal sections were stained with an anti-β-gal antibody to detect LacZ expression. Scale bar, 50 μm. (**e**) The LacZ expression level was significantly reduced in SnAmCyan-labeled neurons co-expressed with LacZ-RNAi, compared to that co-expressed with LacZ-RNAi-Scramble. Box-and-Whisker plot is similar as (**c**). (***) p < 0.001, Brunner-Munzel test.

To achieve sparsely labeled cell-specific manipulation of endogenous gene expression without using a floxed mouse, we modified the Supernova system by combining it with RNA interference (RNAi) technology^26^,^27^. Small hairpin RNA (shRNA) can be transfected into cortical neurons by the IUE-mediated transfection of CAG promotor/microRNA30-based RNAi vector (CAG-LSL-mir30)^14^. By IUE of Supernova-mediated expression vectors carrying shRNA against target genes (details see Methods and Supplementary Table 2), we efficiently reduced their expression level in sparsely labeled cortical neurons (See Fig. 5 and its legend).

### Supernova-mediated TALEN enables sparsely labeled cell-specific gene knockout/editing *in vivo*

**Figure 6:**
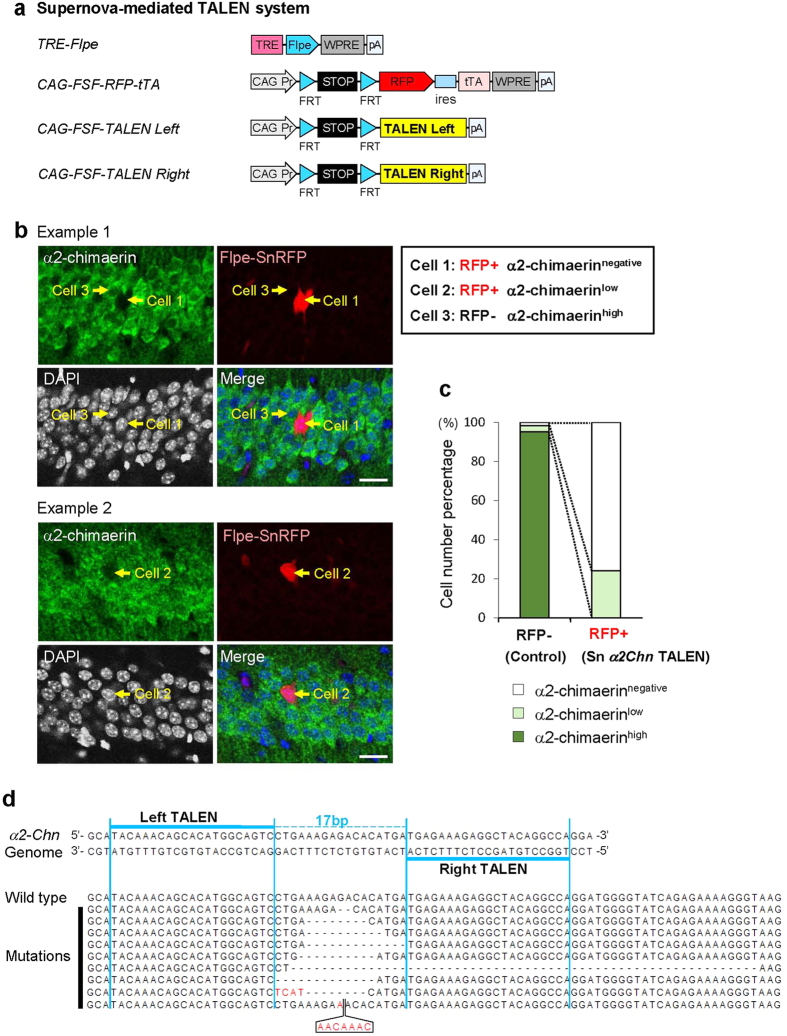
Labeled cell-specific gene knockout in wild-type mouse brain by Supernova-mediated TALEN. (**a**) Schematic representation of a Supernova-mediated TALEN vector set. (**b**) Hippocampal CA1 was introduced with the Supernova *α2-Chn* TALEN vector set. Note that we attenuated SnRFP signal for clear detection of *α*2-chimaerin signal. Two sets of example images were shown. (**c**) Most RFP-negative neurons (control cells) express high level of *α*2-chimaerin protein (*α*2-chimaerin^high^ cell, e.g. Cell 3). While, in majority of RFP-positive neurons [cells expressing Supernova *α2-Chn* TALEN (Sn*α2-Chn* TALEN)], the *α*2-chimaerin protein expression is undetectable (*α*2-chimaerin^negative^ cell, e.g. Cell 1). Only a small portion of RFP-positive neurons shows *α*2-chimaerin protein expression, but very weakly (*α*2-chimaerin^low^ cell, e.g. Cell 2). Control: 1426 cells; Sn*α2-Chn* TALEN: 53 cells. See Experimental Procedures for details. (**d**) Top: wild-type sequence of *α2-Chn* locus. Bottom: mutation patterns detected in Supernova-labeled cells sorted by FACS; red bases: inserted bases; black dashes: deleted bases. Locations of left and right TALENs (thick blue lines) targeting mouse *α2-Chn* gene are shown. Blue dashed lines indicate spacer region (17 bp) between two TALEN target sites. Scale bar: 20 μm (**b**).

To achieve more effective gene suppression, we adapted TALEN-based genome editing technology^28^,^29^ to the Supernova system (Flpe-based version, Fig. 6). To examine the efficiency of Supernova-mediated TALEN, we constructed a pair of TALEN vectors targeting the endogenous *α2-Chn* gene and co-transfected them into the hippocampal CA1 neurons with TRE-Flpe and CAG-FSF-RFP-tTA vectors (Fig. 6a). We used immunohistochemistry to detect *α*2-chimaerin expression. DAPI-staining was performed to visualize cell body location (Fig. 6b). We categorized DAPI-stained cells in the hippocampal CA1 region into three groups depending on the intensity of anti-*α*2-chimaerin signals: *α*2-chimaerin^high^, *α*2-chimaerinn^low^, and *α*2-chimaerin^negative^ (See Materials and Methods and Fig. 6 legend for details). We found that 76.0% ± 6.0% (n = 3 mice) of SnRFP-labeled cells were *α*2-chimaerin^negative^ cells. The rest were *α*2-chimaerin^low^ cells, whereas there were no *α*2-chimaerin^high^ cells. In contrast, *α*2-chimaerin^high^ and *α*2-chimaerin^low^ cells were 95.1% ± 0.5% (n = 3 mice) and 3.2% ± 0.1% (n = 3 mice), respectively, of RFP-negative neurons (Fig. 6c), i.e., in cells that express *α2-Chn*-targeting TALENs, *α*2-chimaerin protein levels were dramatically reduced, suggesting that Supernova-mediated TALEN successfully inhibited *α*2-chimaerin expression in the CA1 pyramidal neurons of wild-type mice.

To confirm the genomic editing via *α2-Chn*-targeting TALENs, we collected fluorescent-labeled cells by performing fluorescence-activated cell sorting (FACS) from the P1 brains that were introduced with the Supernova-mediated *α2-Chn* TALEN vector set using IUE at E14.5. We amplified the target genome locus from a pool of 200 FACS-sorted cells using PCR and cloned into the plasmids (See Experimental Procedures for details). In 13 clones obtained, 12 had mutations (three patterns) and one had wild-type sequence. By repeating the experiments, nine mutation patterns in total were identified (Fig. 6d). These data suggest that Supernova-TALEN-induced genome editing of an endogenous gene is very efficient.

### Supernova-mediated CRISPR/Cas9 enables sparsely labeled cell-specific gene knockout/editing *in vivo*

**Figure 7:**
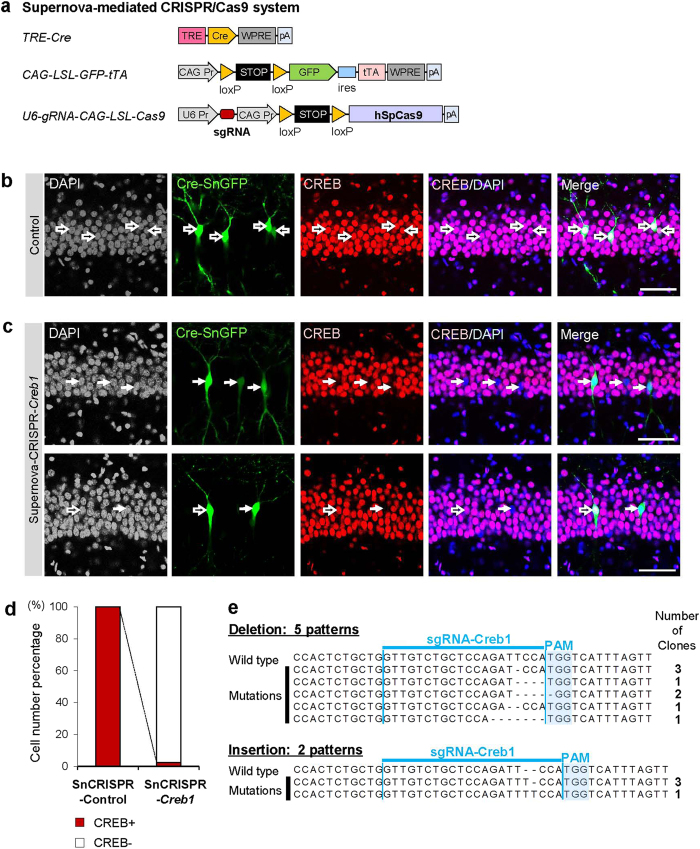
Labeled cell-specific gene knockout in wild-type mouse brain by Supernova-mediated CRISPR/Cas9. (**a**) Schematic for Supernova-mediated CRISPR/Cas9 vector set. (**b,c**) Hippocampal CA1 region of wild-type mice electroporated with Supernova-CRISPR/Cas9 control (without targeting sequence in sgRNA) (**b**) and Supernova-CRISPR/Cas9 targeting mouse *Creb1* (SnCRISPR-*Creb1*) (**c**). CREB, *Creb1* gene product, shows strong and ubiquitous expression in the GFP-negative cells in hippocampal CA1 region (both (**b**,**c**). CREB expression was also detected in all GFP-positive cells that expressed Supernova-CRISPR control (111/111 cells, 3 mice; hollow arrows in (**b**). In contrast, expression of CREB was specifically lacking in almost all GFP-positive cells expressing SnCRISPR-*Creb1* (81/83cells, 3 mice; arrows in (**c**), with a very few exceptions (2/83 cells; hollow arrows, lower panels in (**c**). (**d**) Percentage of numbers of CREB-positive and CREB-negative cells in GFP-positive cells (SnCRISPR-control- or SnCRISPR-*Creb1*-expressing cells). (**e**) Representative mutations found in the *Creb1* locus in GFP-positive cells that were collected from the SnCRISPR-*Creb1*-transfected cortex by FACS at P1. The sgRNA target location is indicated by thick blue lines. The PAM sequence is marked in blue shades. Black dashes: deleted bases. The number of clones showing corresponding mutation pattern was presented in the right. Scale bars, 50 μm (**b**,**c**).

We also combined another genome editing technology CRISPR/Cas9^10^,^16^,^30^ with the Supernova system (Fig. 7 and Supplementary Fig. 9). To evaluate the effectiveness of gene knockout by Supernova-mediated CRISPR/Cas9, we first disrupted the *LacZ* gene in the hippocampal CA1 pyramidal neurons of Rosa-LacZ mice, which ubiquitously express *LacZ* gene (Supplementary Fig. 9a). The vector set comprised CAG-flexed hSpCas9, U6-LacZ-targeting single guide RNA (sgRNA) and Cre-SnGFP, which were used for IUE at E14.5 (Supplementary Fig. 9b). Using immunohistochemistry, we observed that, in the majority of GFP-positive neurons (39/45 cells, three mice), LacZ expression was undetected. In contrast, ubiquitous LacZ expression was confirmed in surrounding unlabeled neurons (Supplementary Fig. 9c).

Next, we assessed the gene editing efficiency of the Supernova-mediated CRISPR/Cas9 system to an endogenous gene in wild-type mice. We chose *Creb1* as the target gene because of its strong and ubiquitous expression in the hippocampus. To reduce the number of vectors used for IUE, we utilized the pX330 vector^30^, which expresses both hSpCas9 and sgRNA from a single vector. The loxP-stop-loxP (LSL) cassette was inserted to create Cre-dependency (Fig. 7a, U6-sgRNA-CAG-LSL-Cas9). We co-transfected it into the hippocampal CA1 pyramidal neurons with TRE-Cre and CAG-LSL-GFP-tTA vectors using IUE at E14.5, and examined CREB protein expression in the P8 mouse hippocampus using immunohistochemistry. We observed that, when the control vector (without targeting sequence in sgRNA) was used, all GFP-positive neurons expressed CREB (111/111 cells, three mice) (Fig. 7b). In contrast, when the vectors carrying sgRNA-targeting *Creb1* (see methods and Supplementary Table 2) were transfected, CREB expression was undetectable in almost all GFP-positive neurons (83/85 cells, three mice) (Fig. 7c,d).

Finally, we confirmed the *Creb1* editing in GFP-positive cells through FACS followed by sequencing analysis on a pool of 300 cells. We successfully identified seven patterns of indel mutations in all 12 clones obtained (Fig. 7e). No wild-type clone was identified. These data indicate that Supernova-mediated CRISPR/Cas9 is a highly effective in achieving single-cell specific gene knockout *in vivo*.

### AAV-based Supernova enables sparse and bright cell-labeling *in vivo*

Virus vector-mediated gene delivery is another powerful approach for gene expression *in vivo*. This method is particularly suitable for labeling of cells in adult animals or cells in the brain regions and organs that IUE-based gene delivery is difficult to be applied for. Adeno-associated virus (AAV) is wildly used for neural labeling, because it is least toxic among available viral vectors, leads to high levels of and long-lasting gene expression, and is safe and easy to engineer^43^,^44^. A certain level of sparseness can be achieved by reducing the copy number of viral vectors. However, simple dilution has a problem that fluorescence signals are often too weak to be used for detailed morphological analyses (Supplementary Fig. 10).

**Figure 8:**
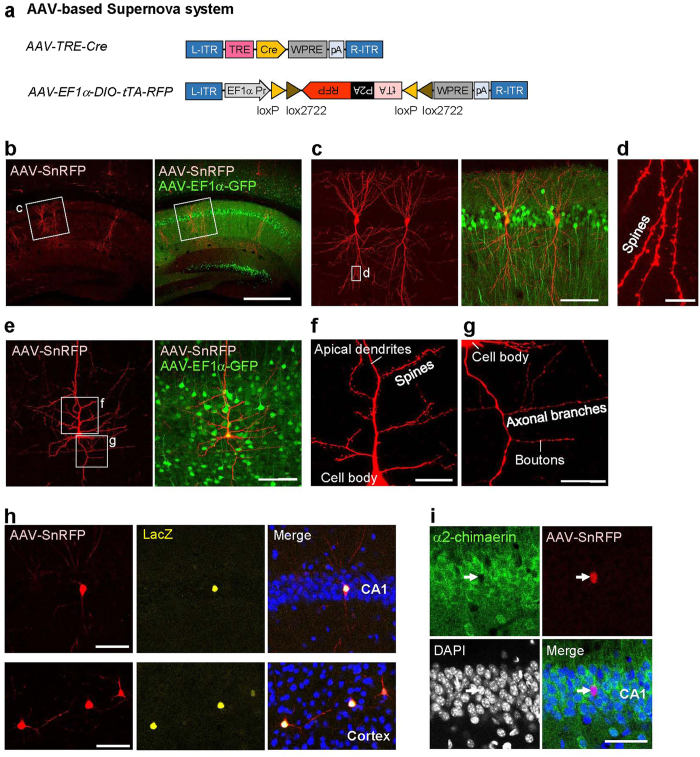
AAV-based Supernova enables sparse and bright neuronal labeling and labeled cell-specific gene knockout *in vivo*. (**a**) Schematic representation of the adeno-associated virus (AAV)-based Supernova vector set, which consists of AAV-TRE-Cre-WPRE (AAV-TRE-Cre) and AAV-EF1*α*-DIO-tTA-P2A-RFP-WPRE (AAV-EF1*α*-DIO-tTA-RFP) vectors. DIO: Double-floxed Inverted Open reading frame. (**b–g**) AAV-based Supernova RFP (AAV-SnRFP) labeled hippocampal CA1 pyramidal neurons (**b–d**) and L5 pyramidal neurons in the cortex (**e–g**) so sparsely and brightly that detailed morphologies such as dendritic spines and axonal boutons of labeled neurons were clearly visible. AAV injection was performed at P10-P13, and brains were fixed at 30 days post-infection (DPI). AAV-EF1*α*-GFP-WPRE (AAV-EF1*α*-GFP) was co-injected as control. (**c**) Higher-magnification images of the squares in (**b**). The rectangle in **c** was further enlarged in (**d)**. (**f,g**) Enlarged images of squares in (**e**). Scale bars, 500 μm (**b**), 100 μm (**c,e**), 10 μm (**d**), 25 μm (**f,g**). (**h**) Cre-mediated genomic DNA recombination detected using RNZ reporter mice was specific to AAV-SnRFP-labeled neurons in the hippocampus (upper panels) and cortex (bottom panels). AAV-SnRFP was injected into the hippocampus or cortex of RNZ mice at P10, and brains were fixed at 40DPI. Coronal sections were prepared and stained with an anti-β-gal antibody, which detects LacZ expression, and DAPI. Scale bars, 50 μm. (**i**) *α*2-Chn was disrupted specifically in an AAV-SnRFP-labeled neuron in the hippocampal CA1. AAV-SnRFP was injected into the hippocampus of *α2-Chn*^flox/flox^ mice at P2, and brains were dissected and sectioned at P18. Immunohistochemistry was performed to detect *α*2-chimaerin expressing cells. *α*2-chimaerin was expressed in most cells (DAPI) but not in an AAV-SnRFP-labeled cell (arrow) in the hippocampal CA1. Scale bar, 50 μm. Note that we partially photobleached AAV-SnRFP signal in these experiments (**h,i**) to avoid high intensity signal of Supernova labeling overwhelms *α*2-chimaerin and LacZ signals.

To achieve bright single-cell labeling via AAV, we constructed the AAV-based Supernova system. To overcome the limitation of the size (<5 kb) of the AAV vector^45^, we used double-floxed inverted open reading frame (DIO)^46^ (Fig. 8a). We injected the AAV-Supernova RFP [AAV-SnRFP: a mixture of AAV-TRE-Cre and AAV-EF1α-DIO-tTA-P2A-RFP-WPRE-PA (AAV-EF1α-DIO-tTA-RFP), see Methods for details] into the hippocampus and deep layers of cortex of P10-P13 mice, and conducted analyses at 30 days post-infection (30DPI) (Fig. 8b–g). We demonstrated that hippocampal CA1 (Fig. 8b–d) and cortical L5 (Fig. 8e–g) pyramidal neurons were sparsely and brightly labeled so that their detailed cellular morphologies were clearly visible (Fig. 8d,f,g). We found that, as early as at 10DPI, the fluorescence signals derived from AAV-SnRFP were already bright (Supplementary Fig. 11). With the AAV-based Supernova, sparseness level was adjustable apparently without affecting the brightness by simply reducing the AAV-TRE-Cre vector concentration (Supplementary Fig. 12).

### AAV-based Supernova enables labeled cell-specific gene knockout *in vivo*

In AAV-based Supernova system, a high level of Cre is expected to be expressed only in sparsely labeled neurons. To examine the efficiency and specificity of Cre-mediated genomic DNA recombination, we injected AAV-SnRFP into hippocampal CA1 regions of Rosa26-loxP-stop-loxP-nlsLacZ (RNZ) reporter mice^47^ at P10, and brains were sampled at 40DPI. We observed that almost all RFP-labeled neurons expressed LacZ (76/80cells, n = 3 mice). Moreover, all LacZ-positive neurons were labeled by AAV-SnRFP (76/76 cells, n = 3 mice) (Fig. 8h, upper panel; Supplementary Fig. 13). Similar results were obtained in the cortex (Fig. 8h, bottom panel). These data clearly indicate that Cre-dependent genomic recombination was highly specific to AAV-SnRFP-labeled neurons.

Next, we characterized the efficiency and specificity of AAV-based Supernova system in knocking-out of endogenous genes. AAV-SnRFP was injected into the hippocampus of *α2-Chn*^flox/flox^ mice at P2, and brains were dissected at P18 (16DPI). In the hippocampal CA1, 97% of RFP-negative cells expressed *α*2-chimaerin (204/210 cells, n = 3 mice), while none of RFP-positive neurons showed *α*2-chimaerin signals (0/20 cells, n = 3 mice) (Fig. 8i). These data suggest high efficiency of AAV-Supernova system in labeled cell-specific gene knockout in floxed mice.

Taken together, Supernova series of vector systems (both IUE- and AAV-based systems) are useful systems that enable both sparse cell-labeling with high fluorescence intensity and labeled cell-specific gene manipulation.

## Discussion

We developed Supernova series of vector systems, which enable single-cell labeling and labeled cell-specific gene manipulation, when introduced by IUE or AAV-mediated gene delivery. Their key features are as follows: First, labeled neurons display a sparse distribution and high fluorescence intensity signal with extremely low background, allowing the analysis of the morphological details of individual neurons. Second, the labeling is applicable for spatially and temporally varying targets and is supposed to have no restriction to specific cell types. Third, sparseness levels are adjustable apparently without affecting brightness levels. Fourth, Supernova enables sparsely labeled cell-specific knockout of any gene in the genome, and in the case of IUE-based system it can be achieved even without using genetically altered animals, such as floxed mice. Finally, a rapid experimental turnover makes Supernova a highly expandable system. In this study, we newly incorporated numerous advanced genetic tools including various fluorescent proteins, site-specific recombination systems, gene manipulation systems (RNAi, TALEN, CRISPR/Cas9), and a vector delivery system (AAV) to improve and expand the Supernova for wider applications.

### Manipulation of a target gene in sparsely labeled cells *in vivo*

Over decades, gene knockout techniques in mice have contributed to our understanding of the molecular mechanisms of neuronal circuit formation and function in mammals^48^,^49^. Later the Cre-mediated conditional knockout strategy using floxed mice was introduced to identify region- and cell type-specific functions of target genes^34^,^35^,^36^,^38^. However, with these approaches, it is generally difficult to distinguish cell-autonomous functions of the gene from cell-non-autonomous ones.

To reveal the molecular mechanisms operating in individual neurons among densely packed and complexly interconnected neurons in the mouse brain, two systems, MADM^1^,^2^ and SLICK^3^, were developed to enable sparse cell labeling and labeled cell-specific gene knockout. MADM is a genetic system utilizing Cre/loxP-mediated interchromosomal somatic recombination between a pair of MADM cassettes knocked-in into specific loci on individual homologous chromosome pairs^1^. It relies on intercrosses among four mouse lines: a pair of MADM knock-in mice, heterozygous mice carrying a target gene null mutation, and a Cre-driver line. Unfortunately, acquiring, breeding, and mating these mouse lines take substantial time, cost, space, and efforts. Moreover, MADM can target only limited number of genes that are located at specific chromosomal loci, such as those distal to Rosa26 locus on chromosome 6^2^. SLICK is a genetic system utilizing two mouse lines, a specific line of SLICK transgenic mice expressing inducible Cre and YFP under the control of two independent Thy1 promoters and floxed mice for a target gene^3^. However, as SLICK relies on unpredictable position-effect variegation of transgenic mouse lines, this system is applicable only for Thy1-expressing neurons in limited brain areas.

Supernova overcomes most of these limitations. First, Supernova experimental turnover (weeks) is much more rapid than that of MADM (years) and SLICK (>months), because Supernova uses IUE or AAV for gene delivery. Second, Supernova is applicable for all genes in the genome. Third, it is applicable for a wide variety of brain areas accessible with IUE or AAV. Fourth, because Supernova vectors use TRE (minimal CMV promoter)^50^ and CAG promoters^51^, they are not restricted to specific cell types. Furthermore and importantly, experimental design utilizing Supernova is highly flexible, and therefore, the optimal conditions (e.g., types of fluorescent proteins for labeling, sparseness levels of labeling) for specific research purpose can be chosen, which is impractical with MADM and SLICK. If cell-type specificity is required, one can design vector sets for the purpose, such as TRE-Flpe and CAG-FSF-LSL-XFP-ires-tTA (in the case of using cell-type specific Cre mouse lines) or TRE-Flpe, Cell-type-specific Promoter-FSF-Dre and CAG-rox-stop-rox-XFP-ires-tTA (in the case of Cre-independent labeling). If high levels of protein expression are inappropriate for a research purpose (such as due to cell toxicity), one can decrease expression levels such as by removing WPRE from the vectors, replacing the CAG promoter with a weaker promoter such as the CMV promoter, and/or just reducing concentration of the CAG-RT-stop-RT-XFP-tTA vector in the mixture.

Here, we present four ways to use Supernova to achieve gene manipulation at a single-cell level: (1) single-cell knockout, by transfecting Cre-based Supernova into floxed mice, for which both IUE and AAV-mediated gene delivery were available; (2) single-cell gene knockdown, by introducing Supernova-RNAi into wild-type mice by IUE; (3,4) single-cell genome editing, by introducing Supernova-mediated TALEN or CRISPR/Cas9 into wild-type mice by IUE.

Before the systematic and detailed characterization of the system in this study, we previously applied the primary version of Supernova, which was Cre-based Supernova-RFP delivered by IUE, to analyze neural circuit refinement in the postnatal somatosensory cortex^18^, which clearly demonstrated the Supernova system potential. When the NMDA receptor NR1 subunit gene is knocked out in all cortical excitatory neurons using the *Emx1*Cre and *NR1*flox mice^34^, many aspects of brain development are impaired, which complicate the phenotypes^34^,^52^,^53^. In contrast, single L4 neuron-specific *NR1* knockout using Cre-based Supernova on *NR1*flox mice reveals the cell-autonomous function of the NMDA receptor in circuit refinement^18^. In addition to these histological analyses, highly sparse and bright labeling of Supernova allows *in vivo* imaging of normal and *NR1* knockout L4 cells, which newly uncovers dendritic dynamics associated with normal and impaired circuit refinement in the neonatal somatosensory cortex^18^.

RNAi is widely used for gene knockdown *in vivo*^12^,^13^,^27^. Recently developed TALEN and CRISPR/Cas9 systems are widely used for gene editing *in vivo*^10^,^12^,^16^. Designing shRNAs, TALENs and CRISPR sgRNAs is simple. These three systems can be applied without using genetically modified animals, such as a floxed mouse. Furthermore, they are also applicable for other species, such as rat, ferret, and marmoset. Therefore, cooperative use of IUE-based Supernova with RNAi, TALEN or CRISPR provides effective ways for the gene functional analysis at single-cell level *in vivo*.

Very recently, the SLENDR (single-cell labeling of endogenous proteins by CRISPR/Cas9-mediated homology-directed repair) system, which is based on IUE, was published^54^ and someone may be confused that SLENDR is similar to the IUE-based version of Supernova. However, these two systems are completely different. SLENDR is an excellent system for protein labeling *in vivo* but not for single-cell labeling or labeled cell-specific gene knockout. In SLENDR system, the sparse labeling and gene knockout are induced independently based on distinct mechanisms (homology-directed repair and non-homologous end-joining, respectively). With SLENDR, gene knockout occurred in about half of the transfected neurons (not sparse), in which some are labeled and others are not (not specific). Similarly, cell labeling occurs in both wild-type and knockout cells (not specific), and therefore antibodies against endogenous proteins are required to distinguish knockout and wild-type cells (expensive, laborious, non-applicable for *in vivo* study). Thus, for the purpose of single-cell gene knockout, which is indispensable for understanding of cell-autonomous function of the gene, using Supernova should be more suitable than that of SLENDR.

### Other applications of Supernova

Apart from single-cell gene knockout, the excellent sparseness and brightness of Supernova enabled many types of researches. Intact neural connectivity was observed at single-cell resolution in optical clearing reagent^33^-treated whole brain (Supplementary Fig. 4), which is valuable for constructing the connectome of the brain. Further, because Supernova enabled multiple gene co-expression in sparsely labeled cells (Fig. 3), following applications are also possible. (1) A fluorescent protein and a calcium indicator co-expression into individual neurons allows simultaneous *in vivo* imaging of neuronal morphology and neural activity by two-photon microscopy. Correlation analysis between the morphological and physiological properties of labeled single neurons during circuit refinement can provide valuable information for understanding activity-dependent neural circuit development. (2) Endogenous protein function in a single cell can be regulated by over-expressing genes encoding dominant negative or constitutively active forms of defined proteins.

### Sparse and bright cell-labeling

To date there are numerous reports for sparse cell labeling using IUE. As an example, combination of low concentration of pCAG-Cre vector and high concentration of pCAG-LSL-XFP vector is reported^22^. This system achieves certain levels of brightness and sparseness but it is often suffered by high background (presence of many darkly labeled cells)^18^,^23^. Another system, PASME^21^, achieves bright cell labeling with low background as the Supernova system does. PASME is applicable for *in vivo* imaging of morphological details of single cortical neurons in the living mouse brain. However, because PASME is the system that relies on *Thy-1* promoter, gene expression is restricted to neurons, in particular, *Thy-1*-expressing neurons. Therefore, its application is limited. On the other hand, because Supernova uses ubiquitous promoters, it is supposed to have no cell-type restriction.

IUE can be applied for various regions, including neocortex, hippocampus, amygdala^55^, striatum^56^, midbrain^57^, dorsal thalamus^58^, hypothalamus^59^, cerebellum^60^, retina^61^ and spinal cord^62^. Despite these, there are also some areas that IUE is difficult to be applied for. For these regions, viral vector-based approaches are indispensable. Several types of virus vectors such as adeno associated virus (AAV)^24^,^63^, rabies virus^19^,^20^, sindbis virus^25^, vesicular stomatitis virus^64^ are also successfully used for sparse cell labeling.

In this study, we also developed AAV-based Supernova system and demonstrated that it labels cells sparsely and brightly as IUE-based Supernova system does. Note that, to our best of knowledges, our AAV-based Supernova is the only viral vector system that achieved both sparse and bright cell labeling and labeled-cell specific gene manipulation *in vivo*. IUE- and virus vector–mediated gene delivery systems have their intrinsic strengths and weaknesses. Therefore, users should choose IUE-based methods or virus-based methods based on their specific research purposes.

Sparse cell labeling is often used for lineage tracing. For the purpose, methods such as reverse-genetics (e.g. MADM) and retrovirus-mediated gene delivery, which involves chromosomal integration of introduced genes, are useful^1^,^6^,^65^. On the other hand, although Supernova-mediated bright cell labeling is maintained long-term (>8 months) in postmitotic neurons (Fig. 2), Supernova appears not suitable for dividing-cell labeling. Because currently available Supernova relies on IUE or AAV for vector delivery, delivered vectors should be diluted and fluorescence intensities should be decreased by cell division.

Altogether, the Supernova system is highly expandable and widely applicable to label a sparse cell population with high fluorescence intensity and essentially no background and to enable simultaneous manipulation of any gene in these labeled cells in higher animals, such as mouse. Thus, the Supernova system is a promising tool to elucidate the molecular and cellular mechanisms underlying various biological processes *in vivo* at a single-cell level.

## Materials and Methods

### Plasmid construction.

#### Cre-based Supernova vectors

- The construction of pK031 (pTRE-Cre) and pK029 (pCAG-loxP-stop-loxP-RFP-ires-tTA-WPRE) was previously described^18^,^50^. Briefly, the PCR-amplified nuclear localization signal (nls)-Cre sequence was ligated into pTRE-Tight vector (Clontech) to obtain pK031. tTA was generated from the pTet-Off-Advanced vector (Clontech) and used for construction of pK029.
- pK038 (pCAG-loxP-stop-loxP-EGFP-ires-tTA-WPRE): The EGFP and ires-tTA fragments were isolated from pEGFP-N1 (Clontech) and pK029 vectors by PCR using primer sets, HM050/HM055 and HM054/HM035, respectively. Obtained two PCR products were combined and re-amplified using outside primers (HM050/HM035) to generate EGFP-ires-tTA. Then the EGFP-ires-tTA fragment was inserted into SalI/NotI sites of the pK029 vector, and the pK038 vector was obtained.
- pK039 (pCAG-loxP-stop-loxP-AmCyan-ires-tTA-WPRE): To construct pK039 vector, the AmCyan fragment, which was excised from the pAmCyan vector (Clontech) by SalI/StuI digestion, was cloned into SalI/EcoRV sites of the pK029 vector.
- pK098 (pCAG-loxP-stop-loxP-nlstagRFP-ires-tTA-WPRE): The encoding sequences of the nls-tagRFP were cloned by PCR from pK022 [TagRFP-N (Evrogen)] with the primer sets: HM079/HM075. The PCR product was ligated into SalI/EcoRV sites of pK038 vector.
- pK102 (pCAG-loxP-stop-loxP-PSD95eGFP-ires-tTA-WPRE): PSD95eGFP was excised with EcoRI and NotI from pCMV-PSD95 GFP (a generous gift from Dr. S. Okabe, Univ. of Tokyo). The product was blunted with Klenow and ligated into blunted SalI/EcoRV site of pK038 vector by replacing EGFP.

#### Flpe-based Supernova vectors

- pK036 (pTRE-Flpe-WPRE): To generate the pK036 vector, the coding sequences of Flpe was amplified from pK016.pCAG-Flpe-ires-Puro (GeneBridge) using the primer pair AY101/AY102. The resulting product was ligated into BamHI/NotI sites of the pK026 [pTRE-Tight vector (Clontech)]. WPRE, excised with NotI from pK029 vector, was ligated into NotI site of obtained TRE-Flpe vector.
- pK037 (pCAG-FRT-stop-FRT-RFP-ires-tTA-WPRE): pK037 vector was generated by replacing loxP-stop-loxP sequence with the FRT-stop-FRT sequence between EcoRI/SalI sites of pK029. FRT-stop-FRT sequence was amplified from the pBS302 vector (Gibco-BRL) using the primer set HM009/HM008.
- pK068 (pCAG-FRT-stop-FRT-EGFP-ires-tTA-WPRE): For the pK068 vector, EGFP-ires-tTA sequence was excised with SalI and NotI from pK038 vector and cloned into SalI /NotI sites of pK037 vector. WPRE, excised with NotI from pK029, was ligated into NotI sites of obtained vector.

#### Dre-based Supernova vectors

- pK073 (pTRE-Dre-WPRE): The Dre sequences were amplified by PCR from pK055 (pCAG-Dre-ires-puro.gcc) vector (a generous gift from Dr. Mitsuru Morimoto, NIG) using primer set WL006/WL007. Resulted product was cloned into the EcoRI/NotI sites of pK036 vector. WPRE, excised with NotI from pK029, was ligated into NotI sites to generate pK073 vector.
- pK129 (pCAG-rox-stop-rox-RFP-ires-tTA-WPRE): For pK129 vector, the rox-STOP-rox was excised with BglII and XhoI from pK078 (pJFRC176-10XUAS-rox-dSTOP-rox-myr::GFP) (a gift from Gerald Rubin, Addgene plasmid #32147) and ligated into BamHI/XhoI sites of pCAGplay vector^66^. Obtained vector was then digested with EcoRI and XhoI and ligated into EcoRI/SalI sites of pK037 vector by replacing FRT-STOP-FRT with rox-stop-rox.
- pK224 (pCAG-rox-stop-rox-EGFP-ires-tTA-WPRE): For pK224, the rox-STOP-rox was excised with BglII and XhoI from pK078 and ligated into BamHI/XhoI sites of pCAGplay vector^66^. EGFP-ires-tTA form the pK068 vector was excised with SalI and NotI and ligated into the XhoI/NotI sites of above obtained vector. WPRE, excised with NotI from pK046 (pCAG-loxP-stop-loxP-RFP-WPRE), was ligated into NotI site of obtained pCAG-rox-stop-rox-EGFP-ires-tTA vector.

#### Supernova-mediated RNAi vectors

- pK177 [pCAG-loxP-stop-loxP-mir30 (GFP RNAi)] and pK178 [pCAG-loxP-stop-loxP-mir30 (GFP RNAi scramble control)]: The shRNA against the coding region of GFP (441–461) and its scramble control were generated by PCR with the template oligonucleotide for GFP shRNA HM084 and the template oligonucleotide for GFP shRNA scramble control HM085, respectively, using the primers HM082/HM083. The amplified products were ligated into XhoI/EcoRI sites of pCAG-loxP-stop-loxP-mir30 vector^14^ (Addgene plasmid #13786). The shRNA sense and antisense target sequences appear in underline in Supplementary Table 2. The insert sequences in the construct were verified by nucleotide sequencing using primers: HM088/HM089.
- pK225 [pCAG-loxP-stop-loxP-mir30 (LacZ RNAi)] and pK226 [pCAG-loxP-stop-loxP-mir30 (LacZ RNAi Scramble control)]: For single cell LacZ knockdown, the shRNA against the coding region of LacZ (651–671) and its scramble control were generated by PCR with the template oligonucleotide for LacZ shRNA WL090 and the template oligonucleotide for LacZ shRNA scramble control WL091, respectively, using the primers HM082/HM083. The sequence for LacZ shRNA scramble control are designed using internet tool termed siRNA Wizard-Generation of scrambled siRNA sequence. The amplified products were ligated into XhoI/EcoRI sites of pCAG-loxP-stop-loxP-mir30 vector^14^. The shRNA sense and antisense target sequences appear in underline. The insert sequences in the construct were verified by nucleotide sequencing using primers: HM088/HM089.

#### Supernova-mediated TALEN vectors

- pK217(pCAG-FRT-stop-FRT-α2 Chn TALEN Left) and pK218 (pCAG-FRT-stop-FRT-α2 Chn TALEN Right): TALENs targeting α2-chimaerin was designed using TALEN Targeter^67^ (https://tale-nt.cac.cornell.edu/). The α2 Chn TALENs were constructed based on the two-step Golden Gate assembly method using the Platinum Gate TALEN Kit (a gift from Takashi Yamamoto, Addgene kit #1000000043). The design of α2 Chn TALEN Left RVD is NI HD NI NI NI HD NI NN HD NI HD NI NG NN NN HD NI NN NG HD. The design of a2 Chn TALEN Right RVD is NN NN HD HD NG NN NG NI NN HD HD NG HD NG NG NG HD NG HD NI. The predicted cut site is at 237^th^ base of mouse α2-chimaerin coding sequence (777 bp). A fragment containing a part of CAG promoter and FRT-stop-FRT was excised with SpeI/SalI from pK037 and ligated to the SpeI/XhoI sites in obtained pK209 (pCMV-a2 Chn TALEN Left) and pK210 (pCMV- a2 Chn TALEN Right).

#### Supernova-mediated CRISPR/Cas9 vectors

- pK232 (pCAG-flex-3 Flag-hspCas9) and pK233 (pX330-U6-gRNALacZ) were kind gifts from M. Yuzaki^12^.
- pK162 is a human codon-optimized SpCas9 and chimeric guide RNA expression plasmid (pX330), which was a gift from F. Zhang (Addgene plasmid #42230).
- pK237 (pU6-Chimeric-CAG-LSL-hspCas9): A fragment containing a part of CAG promoter and loxP-stop-loxP was excised from pK029 with SnaBI and AgeI and inserted into the same sites in pK162 (pX330). pK237 do not carry guide oligo, thus used as a control in Supernova-CRISPR/Cas9 experiment.
- pK238 (pU6-gRNACreb1-CAG-LSL-hspCas9): Guide Oligo WL107/WL108 were annealed and cloned in to the BbsI sites in pK237 according to the Target Sequence Cloning Protocol published by F. Zhang group^68^. The guide RNA target sequence was designed using online tool provided by Feng Zhang’s group (http://crispr.mit.edu/). The genomic sequence NC_000067.6 (18054…18167) from NCBI database was used for blasting.

#### Other constructed IUE-based Supernova vectors

- pK046 (pCAG-loxP-stop-loxP-RFP-WPRE): To generate the pK046 vector, loxP-stop-loxP sequence was amplified from the pK013 [pBS302 vector (Gibco-BRL)] using the primer pair HM010/HM007. The RFP sequence was amplified from the pK023 [pTurboRFP-N (Evrogen)] using the primer pair HM026/HM030. The WPRE sequence was amplified from the pK021[pLenti-Synapsin-hChR2 (H134 R)-EYFP-WPRE] vector^69^ (Addgene) using the primer pair HM019/HM020. Obtained three products were ligated into the EcoRI/SalI, SalI/NotI, and NotI sites of the pCAGplay vector^66^, respectively.

#### AAV-based Supernova vectors

- pK168 (pAAV-EF1α-DIO-RFP-P2 A-tTA-WPRE): The tTA sequence was amplified from the pK029 using primers RY78/RY79. The P2 A-tRFP sequence was amplified from pK025^17^ using the primer pair RY69/RY80. The pK168 was generated by ligation of tTA and P2 A-tRFP into the NheI/BamHI and BamHI/AscI sites of the pK167 (pAAV-EF1α-DIO-EGFP) vector, which was a gift from Karl Deisseroth (Addgene: 27056, Cambridge, MA, USA).
- pK170 (pAAV-TRE-Cre-WPRE): The Cre sequence was amplified from the pK031. The pK170 was generated by ligation of Cre into the EcoRI/BglII sites of the pK169 (pAAV-TRE-MCS-WPRE) vector, which was described elsewhere^70^.

#### Other AAV vectors

- pK165 (pAAV-EF1*α*-RFP-WPRE) is described elsewhere^40^.
- pK229 (pAAV-EF1*α*-EGFP-WPRE): The EGFP sequence was amplified from the pK024 (pCAsalEGFP) using the primer pair WL103/WL104. To generate pK229, the RFP in pK165 (pAAV-EF1*α*-RFP-WPRE) was replaced by EGFP through sub-cloning into EcoRI/BamHI sites.

All coding sequences in each construct were verified by nucleotide sequencing. The constructed plasmids and used primers are summarized in Supplementary Tables 1 and 2, respectively. Request of plasmids should be sent to T.I. (tiwasato@nig.ac.jp).

### Animals

All experiments of animal care and use were conducted according to Guidelines for Proper Conduct of Animal Experiments and were approved by the Institutional Animal Care and Use Committee of the National Institute of Genetics (NIG).

Genotypes of TCA-GFP mice^18^, Emx1 Cre KI-ΔNeo mice^34^,^38^,^39^, CAG-loxP-CAT-loxP-GFP reporter mice (a kind gift of Dr. Jun-ichi Miyazaki)^71^, Rosa26 loxP-stop-loxP-nlsLacZ (RNZ) reporter mice^47^, and α2-Chn floxed mice^41^ were determined by PCR as previously described. Rosa26-nlsLacZ mice were obtained by removing the loxP-stop-loxP cassette in the germline of RNZ reporter mice by Cre-mediated recombination. Wild-type ICR mice were used for evaluating the gene editing efficiencies of Supernova-TALEN and Supernova-CRISPR/Cas9 systems.

Embryos for IUE were obtained by mating one male with one female overnight, and the noon of a day when a vaginal plug was found was designated embryonic day (E) 0.5. The day of birth was counted postnatal day (P) 0.

### In utero electroporation (IUE)

IUE was performed as described previously^18^,^72^. Briefly, timed pregnant mice were anesthetized with sodium pentobarbital (50 mg/kg body weight) in saline. After cleaning the abdomen with 70% ethanol, about 3 cm midline laparotomy was performed and the uterus was taken out. For DNA microinjection, glass capillary tubes (GC150 TF-10; Harvard Apparatus) were pulled using a micropipette puller (PC-10; Narishige). A total of 0.5 μl of DNA solution was injected into the right lateral ventricle of embryos using a mouth-controlled pipette system (Drummond Scientific). Square electric pulses (40 V; 50 ms) were delivered 5 times at a rate of 1 Hz by an electroporator (CUY21 SC; NepaGene, Japan) to embryos through the uterus by holding them with forceps-type electrodes (CY650 P10, NepaGene, Japan). The position of the electrodes for DNA transfection into neurons in the cortex and hippocampus was the same as previous reports^72^. After electroporation, the uterus was repositioned in the abdominal cavity and the abdominal wall and skin were sutured with surgical sutures.

For delivering vectors into layer 5 (L5), L4 and L2/3 cortical neurons, IUE was performed at E13.5, E14.5 and E15.5, respectively. For gene transduction into hippocampal neurons, IUE was performed between E14-E15. Plasmids for IUE were purified with NucleoBond Xtra Midi kit (Macherey-Nagel). The composition of DNA solution for each experiment was summarized as below.

- For Cre-based Supernova labeling, a DNA solution containing the pK031.TRE-Cre (5 ng/μl) and CAG-LSL-XFP-tTA vector (one of pK029, pK038 and pK039) (1 μg/μl) was used. (Supplementary Figs 1, 2a, 5 and 6e–h).
- For Flpe-based Supernova labeling of cortical neurons, a DNA solution containing the pK036.TRE-Flpe-WPRE (5 ng/μl) and CAG-FSF-XFP-tTA vector (either pK037 or pK068) (1 μg/μl) was used (Figs 1 and 2, Supplementary Figs 3, 4, 6a–d and 7b).
- For Flpe-based Supernova labeling of hippocampal CA1 neurons, a DNA solution containing the pK036 (50 ng/μl) and pK037 (1 μg/μl) was used (Supplementary Fig. 2b).
- For Dre-based Supernova labeling, a DNA solution containing the pK073.TRE-Dre-WPRE (5 ng/μl) and CAG-RSR-XFP-tTA vector (either pK129 or pK224) (1 μg/μl) was used (Supplementary Fig. 8).
- For regular labeling experiments, a DNA solution containing the pK024^66^ (1 μg/μl) (Supplementary Fig. 1b right panel, **2a** right panel, **3**, **5**, **6**), or pK025 (1 μg/μl)^17^ (Figs 1c,d, 2 and Supplementary Fig. 1b left and middle panel, **2a** left and middle panel) was used.
- For Flpe-SnGFP and Dre-SnGFP-labeling of cortical neurons in Emx1-Cre KI mice, pK046 (1 μg/μl) was co-transfected as a control (Supplementary Figs. 7 and 8).
- For quantifying co-expression efficiency of Flpe-based Supernova, pK036 (10 ng/μl) together with pK037 (1 μg/μl) and pK068 (1 μg/μl) was used (Fig. 3a). For simultaneous visualization of single-labeled cell and its nucleus, pK031 (10 ng/μl) together with pK038 (1 μg/μl) and pK098 (1 μg/μl) was used (Fig. 3b). For visualizing the dendritic spines in sparse labeled single neurons, pK031 (10 ng/μl) together with pK029 (1 μg/μl) and pK102 (1 μg/μl) was used (Fig. 3c).
- For Supernova-mediated sparse gene knockdown experiments using CAG-loxP-CAT-loxP-GFP reporter Tg mice, a DNA solution containing the pK031 (10 ng/μl), pK098 (1 μg/μl) and pK177 (1 μg/μl) for GFP knockdown or pK178 (1 μg/μl) for control was used (Fig. 5b,c).
- For Supernova-mediated sparse gene knockdown experiments using RNZ mice, a DNA solution containing the pK031 (10 ng/μl), pK039 (1 μg/μl) and pK225 (1 μg/μl) for LacZ knockdown or pK226 (1 μg/μl) for control was used (Fig. 5d,e).
- For examine the efficiency of Supernova-mediated TALEN on disruption of α2-Chn expression in hippocampal neurons, a DNA solution containing the pK036 (50 ng/μl), pK037 (1 μg/μl), pK217 (1 μg/μl) and pK218 (1 μg/μl) was used for IUE at E14.5 (Fig. 6b).
- For confirming the genomic editing via *α2-Chn* TALENs, a DNA solution containing the pK209 (1 μg/μl), pK210 (1 μg/μl) and pK024 (1 μg/μl) or a DNA solution containing the pK036 (400 ng/μl), pK068 (800 ng/μl), pK217 (800 ng/μl) and pK218 (800 ng/μl) was used for IUE at E14.5 to cortex (Fig. 6d).
- For evaluating the effectiveness of gene knockout by Supernova-mediated CRISPR/Cas9 targeting LacZ gene, pK031 (50 ng/μl) together with pK038 (1 μg/μl), pK232 (1 μg/μl) and pK233 (1 μg/μl) was used for IUE at E14.5 to the hippocampus of Rosa-nlsLacZ mice (Supplementary Fig. 9c).
- For assessing the effectiveness of Supernova-mediated CRISPR/Cas9 targeting *Creb1,* a DNA solution containing pK031 (50 ng/μl), pK038 (1 μg/μl) and pK238 (1 μg/μl) was used for IUE at E14.5 to hippocampus in wild-type mice (Fig. 7c). In control experiment, pK162 (1 μg/μl) was used instead of pK238 (1 μg/μl) (Fig. 7b).
- For confirming the *Creb1* modification *via* Supernova-mediated CRISPR/Cas9 system, a DNA solution containing pK031 (50 ng/μl), pK038 (1 μg/μl) and pK238 (1 μg/μl) was used for IUE at E14.5 to cortex in wild-type mice (Fig. 7e).

### Virus preparation

AAV was prepared using AAV-DJ/8 Helper Free Packaging System (Cell Biolabs, San Diego, CA, USA). The pAAV expression vector, together with pAAV-DJ (Agilent Technologies: 240071), pHelper (Agilent Technologies: 240071) was cotransfected into HEK293 T or HEK293 FT cells in Complete Medium with penicillin and streptomycin (DMEM containing 10% heat-inactivated FBS, 1 mM sodium pyruvate solution, 0.075% sodium bicarbonate solution). At 72 h after transfection, the medium containing cell lysate was centrifuged at 2,500 rpm for 30 min. The supernatant was filtered through a 0.45 μm-membrane filter (Millipore, Bedford, MA, USA) and transferred to ultracentrifuge tubes. Two ml of 20% sucrose/PBS was gently added to the bottom of the supernatant. Then, the supernatant was centrifuged at 22,000 rpm at 4 °C for 2 hours in a Beckman ultracentrifuge. The virus pellet was resuspended in 100 μl cold PBS, aliquoted and stored at −80 °C. The genomic titer of each virus was determined by quantitative PCR using Power SYBR Green PCR Master Mix (Applied Biosystems, Warrington, UK), the primer pair WL123/WL124 and Thermal Cycler Dice Real Time System TP800 (Takara Bio Inc, Tokyo, Japan). The viral samples were treated with RNase-free DNase I (Takara Bio Inc, Tokyo, Japan) at 37 °C for 30 min. DNase I was inactivated by incubation at 65 °C for 10 min. The standard curve was made by using 10-fold serial dilution of pK165 plasmid (10–10^8^ copies), which was digested with EcoRI and purified in advance.

### Virus injection

P10-P13 mice were anesthetized with 1.7–2.5% isoflurane. The skin on injection point was opened. A hand drill (MINIMO, Tokyo, Japan) was used to drill the skull. Viral were injected unilaterally into the left hippocampus or cortex. During the virus injection, the dose of isoflurane was then reduced to 1.5%. The injection site for labeling neurons in hippocampal CA1 regions and deep cortical layers was located approximately 1 mm lateral to the sagittal suture, half between the lambda and bregma, and −1.5~−0.8 mm from the dural surface. For viral infection in P2 pups, the injection site was 1.5 mm left to lambda, −1.5~−1.0 mm from the surface. Virus delivery was performed at a rate of 200 nl per min using a programmable syringe pump (KD Scientific) with 34-gauge beveled NanoFil needle (World Precision Instruments, Sarasota, FL, USA). Virus vectors used for injection in this study are summarized as follows:

For regular AAV labeling, AAV-EF1*α*-RFP-WPRE (5.5 × 10^11^ genome copies/ml) was diluted 10–10^3^ folds in PBS. Then, equal amount of AAV-EF1a-RFP-WPRE with/without dilution, AAV-EF1a-GFP-WPRE (3.5 × 10^11^ genome copies/ml) and PBS was mixed. For one mouse, 1000 nl viral mixture was injected (Supplementary Fig. 10).

For AAV-SnRFP labeling, AAV-TRE-Cre-WPRE (2.3 × 10^13^ genome copies/ml) was diluted 10^2^–10^6^ folds in PBS. Equal amount of AAV-TRE-Cre-WPRE with/without dilution, AAV-EF1*α*-DIO-tTA-RFP (1.7 × 10^13^ genome copies/ml) and AAV-EF1a-GFP-WPRE (3.5 × 10^11^ genome copies/ml) was mixed and used for injection (Supplementary Fig. 12). In Fig. 8b–g, AAV-TRE-Cre-WPRE with 10^5^ fold dilution was used. In Supplementary Fig. 11, AAV-TRE-Cre-WPRE with 10^4^ fold dilution was used. For one mouse, 1000 nl viral mixture was injected.

For AAV-SnRFP injection in RNZ mice, AAV-TRE-Cre-WPRE (2.3 × 10^13^ genome copies/ml) was diluted 10^5^ fold in PBS and mixed with same amount of AAV-EF1*α*-DIO-tTA-RFP (1.7 × 10^13^ genome copies/ml). 1000 nl viral mixture per mouse was used for injection (Fig. 8h, Supplementary Fig. 13).

For AAV-SnRFP injection in *α*2-Chn floxed mice, AAV-TRE-Cre-WPRE (2.3 × 10^13^ genome copies/ml) was diluted 10^4^ fold in PBS, mixed with same amount of AAV-EF1*α*-DIO-RFP-tTA (1.7 × 10^13^ genome copies/ml). 300 nl viral mixture was used for injection in one P2 pup (Fig. 8i).

### Tissue processing and immunohistochemistry

For histological analysis, mice were euthanized using an overdose of isoflurane anesthetic and followed by quick decapitation with surgical scissors. Pregnant mice were euthanized by cervical dislocation. Mouse embryos were surgically removed and euthanized by quickly decapitating with scissors. For immunohistochemistry and SeeDB procedure, mice were euthanized under anesthesia with an intraperitoneal injection of pentobarbital and transcardially perfused with 10–30 ml of saline, followed by 20 ml of 4%paraformaldehyde (PFA) in 0.1 M phosphate buffer (PB) pH7.4. Brains were immersed into 4%PFA in 0.1 M PB at 4 °C for overnight and then transferred into 30% sucrose in 0.1 M PB and store at 4 °C for 2 days.

For neural labeling experiment, brains were cut on a freezing microtome (ROM-380; Yamato) into coronal sections at 100 μm. Sections were rinsed in 0.1 M PB and mounted with VECTASHIELD Mounting Medium or 0.02% n-propyl gallate in 90% glycerol.

Immunohistochemistry of GFP, β-galactosidase (β-gal), and CREB were done on 50 μm or 60 μm coronal sections. Briefly, sections were first permeabilized and blocked in 0.2% Triton X-100/5% normal goat or donkey serum (Sigma) in 0.1 M PB for 1 hour at room temperature and then incubated at 4 °C for overnight with primary antibodies. Next day, sections were rinsed in 0.1 M PB and incubated with secondary antibodies in at 4 °C for overnight. For GFP immunohistochemistry, anti-GFP rat antibody (1:1000, Nacalai Tesque #04404–84) and Alexa 488 Donkey anti-rat IgG (H+L) (1:1000, Invitrogen) was used (Fig. 5b). β-gal immunohistochemistry was performed to detect the LacZ expression. Anti-β-gal mouse antibody (1:1000, Promega #Z378 A) and Alexa 568 goat anti-mouse IgG (1:1000, Invitrogen) (Fig. 5d and Supplementary Fig. 9a,c) or Alexa 647 goat anti-mouse IgG (1:1000, Invitrogen) (Fig. 8h, Supplementary Fig. 13a,b) was used. For CREB immunohistochemistry, anti-CREB(48 H2) Rabbit mAb (1:1000, Cell Signaling Technology, #9197) and Alexa 568 goat anti-Rabbit IgG(H+L) (1:1000, Invitrogen) (Fig. 7). Sections were rinsed in 0.1 M PB and further stained with DAPI (Roche, USA) for nuclear staining if necessary. After that sections were mounted with VECTASHIELD Mounting Medium or 0.02% n-propyl gallate in 90% glycerol.

Immunohistochemistry of α2-chimaerin were done on 40 μm coronal sections sectioned frontally (Figs 4b, 6b and 8i). Briefly, sections were rinsed in 0.1% Triton X-100 in 1 × PBS (PBT) twice and incubated in 3% H_2_O_2_/PBS at room temperature for 20 min. Then, sections were treated with pepsin (0.1 mg/μl) in 0.2 M HCl/0.1 M PB for 7 minutes at 37 °C. Next, sections were rinsed in PBT for three times and incubated with 10% normal goat serum (NGS) in PBT at room temperature for 1 hour. After blocking, sections were incubated with anti-α2-chimaerin antibody^42^ (1: 20,000 dilution)/3% NGS in PBT^42^ at 4 °C for overnight. Next day, sections were rinsed with PBS for three times and incubated in biotinylated goat anti-rabbit IgG (Vectastain Elite PK-6101)/3% NGS in PBS (250 μl/well) at 4 °C for overnight. Then, sections were rinsed with PBS and incubated in StrABC/HRP (250 μl/well) at room temperature for 1 hour with shaking. Before the detection, sections were blocked in 10% NGS in PBT at room temperature for 30 min with shaking. After incubation in Fluorophore Amplification Reagent working solution for 8 min at room temperature, sections was washed and further stained with DAPI (Roche, USA) for nuclear staining. Sections were mounted with VECTASHIELD or 0.02% n-propyl gallate in 90% glycerol.

### *In vivo* imaging

Two-photon *in vivo* imaging was performed as described previously with minor modification^18^. Briefly, neonate was anesthetized with isoflurane. For analgesic and anti-inflammation, carprofen (Rimadyl, Zoetis) (5 mg/kg) was subcutaneously injected. Skin covering the skull was removed using scissors to expose skull followed by applying of Vetbond (3 M) to fix and stop bleeding. Then, a small piece of bone covering the Supernova-labeled area was removed with a sterilized razor blade, keeping the dura intact. In order to keep the brain moist, cortex buffer (125 mM NaCl, 5 mM KCl, 10 mM glucose, 10 mM Hepes, 2 mM CaCl_2_, and 2 mM MgSO4; pH 7.4) was applied during surgery. After that, the window was covered with 1% low melting point agarose (Sigma) in cortex buffer and a custom-made round cover glass (diameter: 3 mm) (Matsunami). A custom-made titanium bar was attached near the window. The dental cement was applied to secure the exposed region. After recovery, the pup was anesthetized with 1.3% isoflurane and fixed to microscope stage using titanium bar. During imaging, the dose of isoflurane was then reduced to 0.9%–1.0%. Heating pad was used to keep pup warm. *In vivo* imaging was performed using a LSM 7 MP multiphoton microscope (Zeiss) with a HighQ-2 laser (Spectra-Physics) running at 1045 nm. The fluorescent signal of Supernova-labeled cortical neurons was captured using a BiG detector (Zeiss) with filters (500–550 nm and 575–620 nm) and 20 × objective lens (1.05 NA). Sequential z-images consisted of optical sections (512 × 512 pixels; 1.18 μm/pixel) with 1.66 μm intervals. For reconstruction of the cellular morphology, IMARIS Filament Tracer software (Bitplane) was used.

### Optical clearing using SeeDB

Perfused and fixed brains were cleared with the SeeDB according to the standard protocol described previously^33^. In brief, the brains were serially incubated in 20 ml of 20%, 40% and 60% (weight/volume) fructose in distilled water, each for 8 hours in 50 ml conical tubes with overhead tube rotator at 4 rpm at 25 °C. Samples were then incubated in 80% fructose for 12 hours, 100% fructose for 12 hours and finally in SeeDB containing 80.2% weight/weight fructose for 24 hours with rotation at 25 °C. All fructose solutions contained 0.5% *α*-thioglycerol.

### Two-photon imaging of SeeDB-treated samples

For imaging, SeeDB-cleared whole brain samples were put into a Tissue-TEK II (No. 2, Sakura Finetek, Japan) and covered by a micro cover glass (24 × 60 mm, thickness No.1 0.12–0.17 mm, Matsunami, Japan). The SeeDB solution was used for immersion (Supplementary Fig. 4). Imaging was performed using a LSM 7 MP multiphoton microscope (Zeiss) with a HighQ-2 laser (Spectra-Physics), a BiG detector, and a 20× objective lens (1.05 NA) from the dorsal cortical surface. RFP in callosal projection neuron were excited, and emitted fluorescence was filtered (575–620 nm). Sequential z-images consisted of optical sections (512 × 512 pixels; 1.19 μm/pixels) with 1.74 μm intervals. Laser power was manually adjusted to give constant fluorescent intensities at all depths. The whole cellular morphology of single L2/3 callosal projection neuron was reconstructed from totally 23 × 1 blocks (1block = 607.28 μm × 607.28 μm, Z_max_ = 1.5 mm from the surface) images from the electroplated side to contralateral side where the axons projected. Acquired three-dimensional images were analyzed using the IMARIS Filament Tracer software (Bitplane).

### Image analysis and quantification

Fluorescent images were acquired using a TCS SP5 confocal microscope (Leica).

Basically, for obtaining the whole view of labeled neurons, target region was imaged as z-stacked-images consisted of optical sections (1024 × 1024 pixels) with 7 μm intervals using 10 × objective lens (0.40 NA). For visualizing the fluorescent-labeled neurons, Z-stacked-images consisted of optical sections (1024 × 1024 pixels, physical length 387.50 × 387.50 μm) with 1.20 μm intervals using 40 × objective lens (0.85 NA) was obtained. For visualizing dendritic spines and axonal boutons of Supernova-labeled neurons, sequential z-images consisted of optical sections (1024 × 1024 pixels) with 0.5 μm intervals using 63x oil immersion objective (1.4 NA) with zooms was imaged.

For quantification of sparseness of Supernova labeling, high laser power was used to ensure that all labeled cell can be detected. Sparseness levels were quantified as the percentage of the number of Supernova-visualized neurons to the number of transfected neurons, which was labeled by CAG-GFP or CAG-RFP for normalizing the transfection efficiency (Fig. 2, Supplementary Figs 3 and 6).

For comparing the fluorescent intensity in the somata of neurons that were labeled by Flpe-SnRFP and Flpe-SnRFPΔtTA, fluorescent images were acquired using a confocal microscope under low laser power to avoid the saturation of fluorescent signals in somata of labeled neurons. Due to the great difference of the RFP signal intensity between the two groups, acquisition parameters optimized for detecting RFP signals in Flpe-SnRFPΔtTA-labeled neurons often led the saturation of RFP signals in Flpe-SnRFP-labeled neurons. The maximum intensity of RFP signals in the cell body of labeled was used for making cumulative plot in Supplementary Fig. 3f, RFP signal intensity = 255 grey values represent saturation.

For examination of the genomic recombination efficiency of Cre-based Supernova system in floxed mouse lines, Cre-SnRFP was electroporated (Fig. 4b) or AAV-SnRFP (Fig. 8i) was injected into hippocampal CA1 region in *α2-Chn*^flox/flox^ mouse. The whole view of hippocampus was imaged as z-stacked-images consisted of optical sections (1024 × 1024 pixels) with 7 μm intervals using 10 × objective lens (0.40 NA) (Fig. 4b upper panels). The images used for quantification was one-step image (no z-stack) (2048 × 2048 pixels) that taken by 40 × objective lens (0.85 NA) (Fig. 4b bottom panels and Fig. 8i). Because we observed that the α2-chimaerin signal intensity, which are determined by immunohistochemistry, were varied between sections and procedures. Thus, we chose RFP-negative cells that surround Supernova-labeled cells in the same section (Fig. 4) or RFP-negative cells in un-injection side in the same section (Fig. 8i) as the control cells. We counted the percentage of cells that show α2-chimaerin signal to the cells visualized by DAPI staining in both Cre-SnRFP-labeled RFP-positive cells and RFP-negative cells. We also quantified the α2-chimaerin signal intensity in Cre-SnRFP-labeled cells and RFP-negative cells in α2-Chn^flox/flox^. The lowest raw intensity (gray value) of α2-chimaerin signal in Cre-SnRFP-labeled cell in α2-chimaerin^flox/flox^ mice was considered as the background. Normalized α2-chimaerin signal equals raw α2-chimaerin intensity (gray value) minus background (Fig. 4d).

For quantification of the efficiency of RNAi-mediated gene knockdown in Supernova-labeled neurons, Z-stacked-images consisted of optical sections (1024 × 1024pixels) with 1.20 μm intervals using 40 × objective lens (0.85 NA) was used (Fig. 5b,d). The raw intensity of reporter gene expression detected by immunohistochemistry was plotted (Fig. 5c,e). To avoid experimental deviation, IUE, sampling, sectioning and immunohistochemistry of all samples (both scramble control-group and RNAi group) were simultaneously performed.

For the experiment of TALEN-mediated gene manipulation using Supernova system, one-step image (no z-stack) (2048 × 2048 pixels) that was taken by 40 × objective lens (0.85 NA) was used for quantification (Fig. 6b). The cells in each image were divided into three groups depending on intensities of α2-chimaerin signals. Because hippocampal CA1 pyramidal neurons ubiquitously express very high levels of α2-chimaerin during early postnatal period, thus α2-chimaerin^high^ cells refer to cells that show high intensity of α2-chimaerin signals, and the intensity of the signal are similar with that in surrounding RFP-negative cells (control cells). α2-chimaerin^negative^ refers to cells in which the α2-chimaerin signals were undetected. α2-chimaerin^low^ cells refer to cells in which the α2-chimaerin signals were detected but their intensities were weaker than those in surrounding RFP-negative cells (control cells). The percentage of α2-chimaerin^high^, α2-chimaerin^low^ and α2-chimaerin^negative^ in SnRFP-labeled cells and RFP-negative cells (control cells) were counted (Fig. 6b,c).

For examining the effectiveness of Supernova-mediated CRISPR/Cas9 system, Supernova-CRISPR/Cas9 targeting LacZ was introduced into cells in hippocampal CA1 in RNZ-LacZ mouse (Supplementary Fig. 9). LacZ expression in the P5 mouse brain was visualized by immunohistochemistry using anti-β-gal mouse antibody (1:1000, Promega #Z378 A) and imaged as tilescan image (2048 × 2048 pixels) using 5 × objective lens (0.15 NA) (Supplementary Fig. 9a). The enlarged images of CA1 regions were captured as one-step image (no z-stack) (2048 × 2048 pixels), which was taken by 40 × objective lens (0.85 NA) (Supplementary Fig. 9c). The focus was adjusted according to the round shape of DAPI-visualized nucleus that with longest diameter. Only the cell on the focus was analyzed.

For examination of the efficiency of Supernova-CRISPR/Cas9 targeting *Creb1,* we verified the expression pattern of CREB in the P8 mouse brain by immunohistochemistry using anti-CREB (48 H2) rabbit mAb (1:1000, Cell Signaling Technology, #9197). For quantification, the images of hippocampal CA1 regions were taken as one-step image (no z-stack) (2048 × 2048 pixels), which was taken by 40 × objective lens (0.85 NA) (Fig. 7b,c). The focus was adjusted as above described.

### Fluorescence-activated cell sorting (FACS) and sequencing

Six days after IUE-mediated delivery of Supernova-TALEN or Supernova-CRISPR/Cas9 vectors, GFP-positive regions of the cortex were quickly dissected from the brains (at P1) and transferred immediately to pre-warmed HBSS (Gibco, Invitrogen, #24020). One hour after TrypLE^TM^ Express (Gibco, Invitrogen, #12605) treatment at 37 °C, cortical neurons were dissociated in PBS(−) buffers and washed with FACS buffer [PBS(−) containing 0.02%FBS] for three times. FACS was performed using a JSAN (Japan-made sorter, analyzer) desktop cell sorter. GFP-positive cells were sorted in FACS buffer, centrifuged at 5000 rpm at 4 °C for one hour and stored at −20 °C until use. Genomic DNA locus including TALEN or CRISPR/Cas9 gRNA target sites was amplified by PCR using KOD plus (TOYOBO, Japan) with following primers: WL105/106 for TALENs targeting *α2-Chn*, WL111/112 for CRISPR/Cas9 targeting *LacZ*; WL113/114 for CRISPR/Cas9 targeting *Creb1*. Obtained PCR products were cloned into TOPO vector (Invitrogen) and followed by sequencing using M13 F, M13 R or T7 primers. The sequence alignment was done by CLC Sequencing Viewer 7 (CLC bio, Aarhus, Denmark).

### Statistics

All values represent mean ± standard errors of the mean (SEM). Microsoft Excel 2013, Python 3.5.1 and R version 3.2.5 were used to conduct the statistical analyses. The normal distribution of the data was analyzed by Shapiro-Wilk Test. p < 0.05 was considered as non-normal distribution. The significance of the differences was assessed using Welch’s t-test for two independent samples. p < 0.05 was considered significant. For non-normal distribution data, the significance of the differences was assessed using Brunner-Munzel Test for two independent samples. P < 0.05 was considered significant. Asterisks in figures indicate significance as follows: *p < 0.05, **p < 0.01, ***p < 0.001.

## Additional Information

**How to cite this article**: Luo, W. *et al*. Supernova: A Versatile Vector System for Single-Cell Labeling and Gene Function Studies *in vivo*. *Sci. Rep.* **6**, 35747; doi: 10.1038/srep35747 (2016).

## Supplementary Material

Supplementary Information
