# Supplementary material for: Supernova: A Versatile Vector System for Single-Cell Labeling and Gene Function Studies in vivo
Source: Sci Rep. 2016 Oct 24;6:35747. doi: 10.1038/srep35747 (PMC5075795; doi:10.1038/srep35747)
Supplement: Supplementary Information [file srep35747-s1.pdf]

Supplementary Information

Supernova: A Versatile Vector System for Single-Cell Labeling and Gene Function Studies in vivo

Wenshu Luo, Hidenobu Mizuno, Ryohei Iwata, Shingo Nakazawa, Kosuke Yasuda, Shigeyoshi Itohara and Takuji Iwasato

Supplementary Information Inventory

Supplementary Figures 1-13

Supplementary Tables 1-2

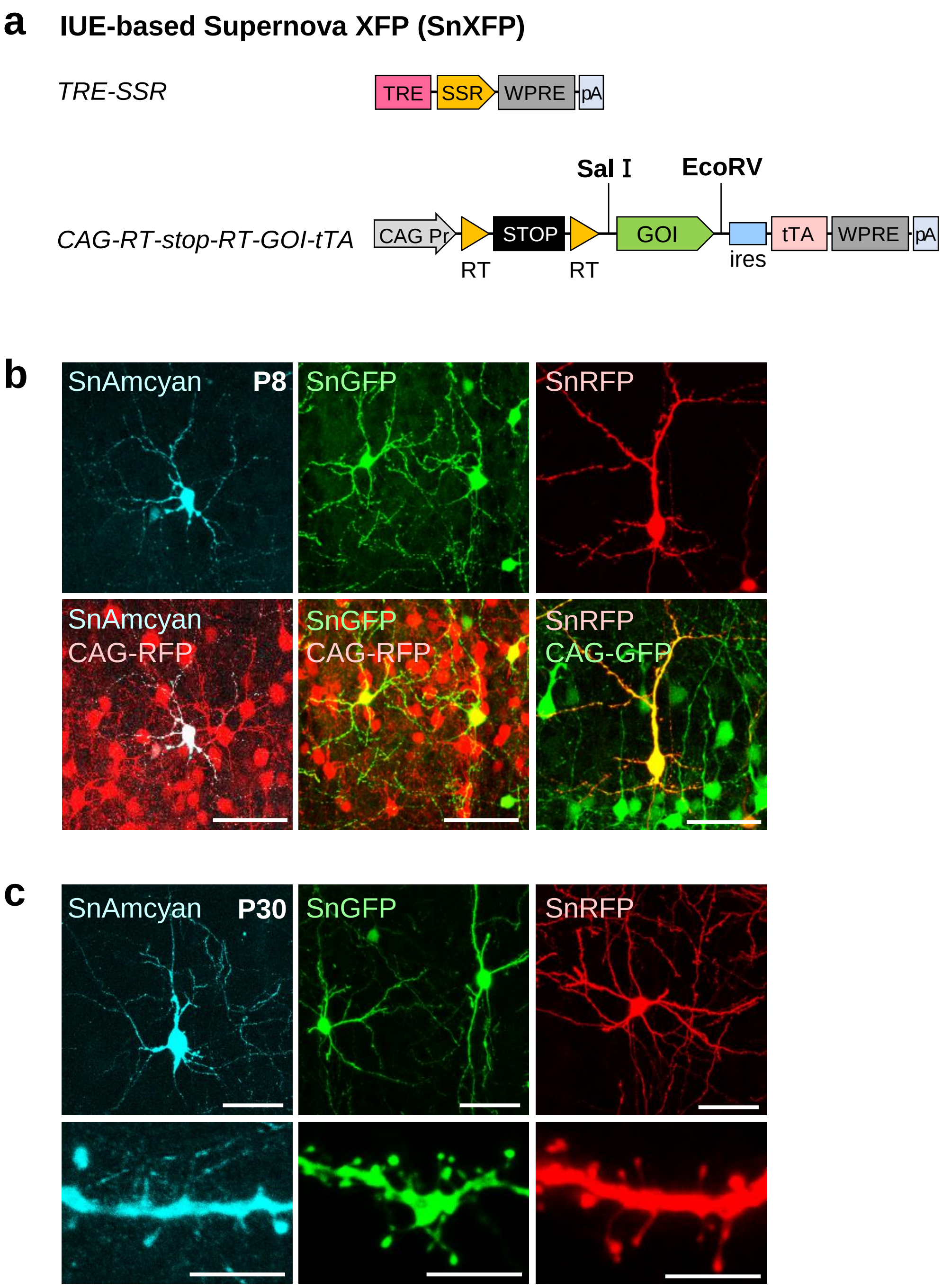
a
IUE-based Supernova XFP (SnXFP)
TRE-SSR
TRE
SSR
WPRE
pA
CAG-RT-stop-RT-GOI-tTA
Sal I
EcoRV
CAG Pr
STOP
GOI
tTA
WPRE
pA
RT
RT
ires
b
SnAmcyan
P8
SnGFP
SnRFP
SnAmcyan
CAG-RFP
SnGFP
CAG-RFP
SnRFP
CAG-GFP
c
SnAmcyan
P30
SnGFP
SnRFP

Supplementary Figure 1. A fluorescent protein can be replaced with another protein easily in Supernova system

(**a**) Restriction sites (SalI/EcoRV) were introduced into the CAG-RT-stop-RT-GOI-tTA vector[18]. Therefore, any gene of interest (GOI; e.g., GFP and RFP) can be easily subcloned into this vector.

(**b**) Cortical neurons sparsely labeled with various fluorescent proteins by the Cre-based Supernova system [Supernova-AmCyan (SnAmCyan), Supernova-GFP (SnGFP) and Supernova RFP (SnRFP)]. CAG-GFP or CAG-RFP was introduced together to label transfected cells. Scale bars, 50 μm.

(**c**) High intensity of Supernova labeling enabled clear visualization of dendritic spines. Scale bars: 50 μm (upper); 5 μm (lower).

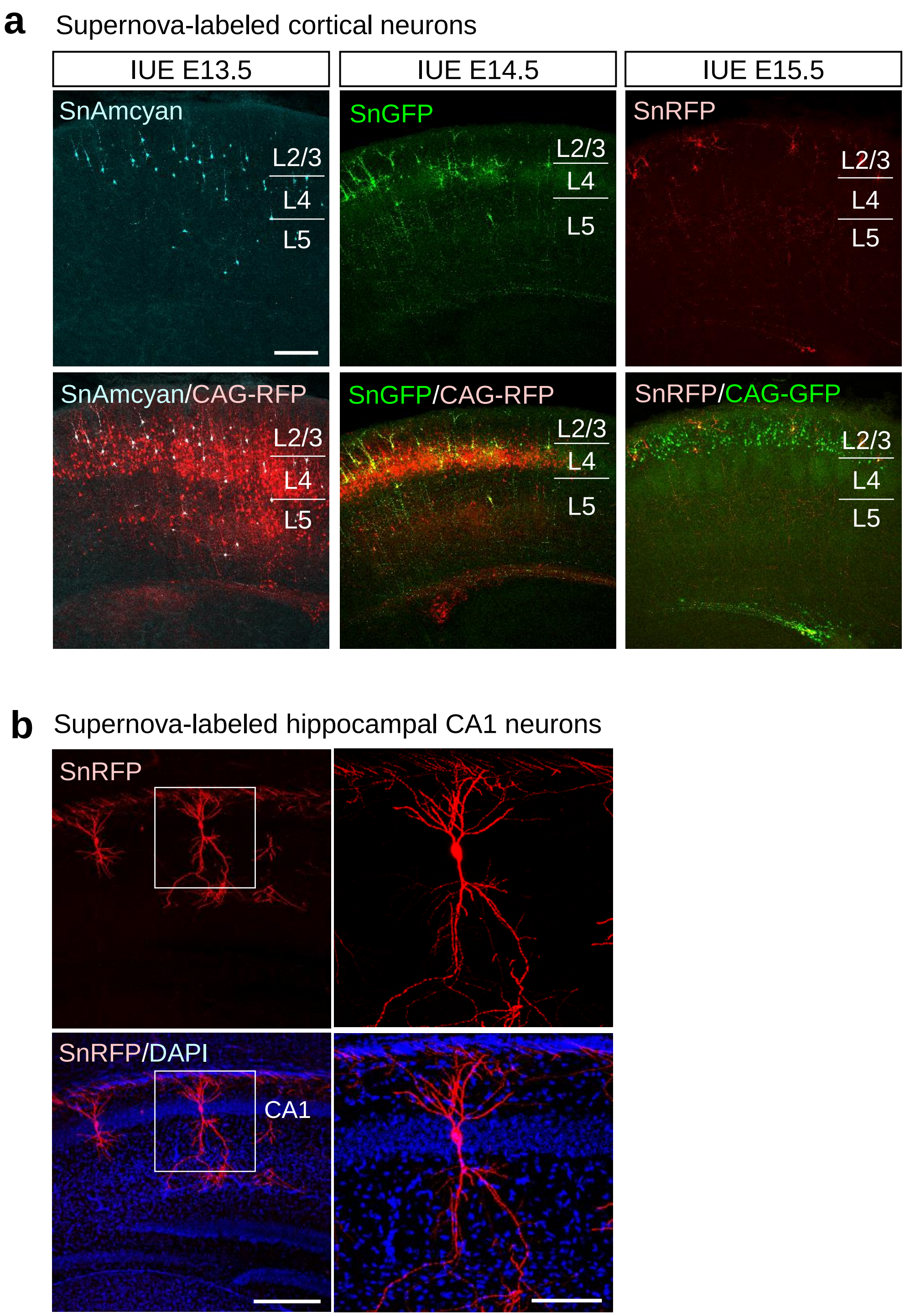
a
Supernova-labeled cortical neurons
IUE E13.5
IUE E14.5
IUE E15.5
SnAmcyan
L2/3
L4
L5
SnGFP
L2/3
L4
L5
SnRFP
L2/3
L4
L5
SnAmcyan/CAG-RFP
L2/3
L4
L5
SnGFP/CAG-RFP
L2/3
L4
L5
SnRFP/CAG-GFP
L2/3
L4
L5
b
Supernova-labeled hippocampal CA1 neurons
SnRFP
SnRFP/DAPI
CA1

Supplementary Figure 2. IUE-based Supernova is applicable to various brain regions.

(**a**) IUE-mediated Supernova enabled sparse labeling of cortical neurons. Supernova AmCyan (SnAmCyan) was delivered to layers 2-5 (L2-L5) by in utero electroporation (IUE) at embryonic day 13.5 (E13.5). SnGFP and SnRFP were delivered into L4 and L2/3 cortical neurons by IUE at E14.5 and E15.5, respectively. Cre-based Supernova was used in these experiments. CAG-GFP or CAG-RFP vector was co-electroporated to indicate transfected neurons. Scale bars, 250μm.

(**b**) Supernova can also be adapted to the hippocampus. Flpe-based SnRFP was introduced into hippocampal CA1 pyramidal neurons by IUE at E14.5. Hippocampal slice was prepared at P14. Right: higher-magnification images of the square in the left panel. Scale bars: 250 μm (left); 100 μm (right).

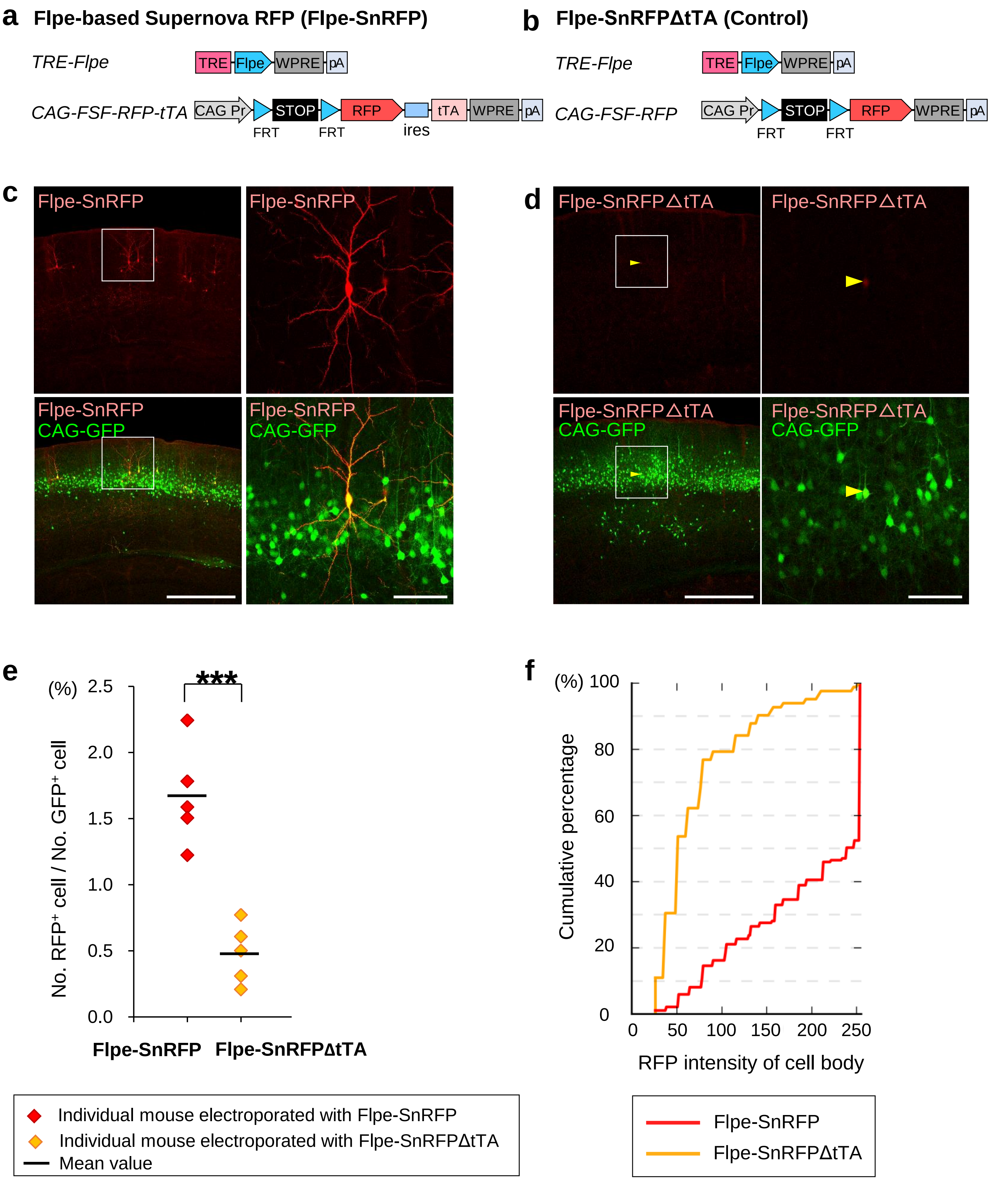
a Flpe-based Supernova RFP (Flpe-SnRFP)
TRE-Flpe
TRE Flpe WPRE pA
CAG-FSF-RFP-tTA
CAG Pr STOP RFP tTA WPRE pA
FRT FRT ires
b Flpe-SnRFPΔtTA (Control)
TRE-Flpe
TRE Flpe WPRE pA
CAG-FSF-RFP
CAG Pr STOP RFP WPRE pA
FRT FRT
c
Flpe-SnRFP
Flpe-SnRFP
Flpe-SnRFP CAG-GFP
Flpe-SnRFP CAG-GFP
d
Flpe-SnRFPΔtTA
Flpe-SnRFPΔtTA
Flpe-SnRFPΔtTA CAG-GFP
Flpe-SnRFPΔtTA CAG-GFP
e
(%)
2.5
2.0
1.5
1.0
0.5
0.0
***
No. RFP+ cell / No. GFP+ cell
Flpe-SnRFP
Flpe-SnRFPΔtTA
Individual mouse electroporated with Flpe-SnRFP
Individual mouse electroporated with Flpe-SnRFPΔtTA
Mean value
f
(%) 100
80
60
40
20
0
Cumulative percentage
0 50 100 150 200 250
RFP intensity of cell body
Flpe-SnRFP
Flpe-SnRFPΔtTA

Supplementary Figure 3. tTA/TRE cycles significantly enhanced RFP expression in SnRFP-labeled neurons.

(**a**) Schematics showing Flpe-based Supernova RFP (Flpe-SnRFP).

(**b**) As a control for examining the efficacy of tTA/TRE enhancement, Flpe-SnRFPΔtTA (TRE-Flpe and CAG-FSF-RFP) was used. CAG-FSF-RFP vector was generated by removing ires-tTA from the CAG-FSF-RFP-tTA vector.

(**c,d**) L4 cortical neurons labeled with Flpe-SnRFP (**c**) and Flpe-SnRFPΔtTA (**d**). The CAG-GFP vector was co-electroporated to mark the transfected neurons. Right: higher-magnification of the square in the left panel. Arrowhead: A L4 cortical neuron darkly labeled by Flpe-SnRFPΔtTA. Scale bars: 500 μm (left panel); 100 μm (right panel)

(**e**) In the Flpe-SnRFPΔtTA-transfected cortex, RFP-labeled cells were barely found. The ratio of RFP-positive cell number to GFP-positive cell (transfected cell) number in Flpe-SnRFPΔtTA-transfected cortex (0.5%±0.1%, n=5 mice) was significantly lower than that in Flpe-SnRFP–transfected cortex (1.7%±0.2%, n=5 mice). All values represent as mean ± SEM. (***) $p < 0.001$, Welch's *t*-test.

(**f**) In the Flpe-SnRFPΔtTA-transfected cortex, barely found RFP-labeled cells were very dark. In contrast, in the Flpe-SnRFP-transfected cortex about half of labeled neurons are very bright showing saturated RFP signals (raw RFP intensity = 255 grey values). Because Flpe-SnRFP-labeling was much brighter than Flpe-SnRFPΔtTA-labeling, acquisition parameters optimized for RFP signals in Flpe-SnRFPΔtTA-labeled neurons usually led the saturation of RFP signals in Flpe-SnRFP-labeled neurons (85 cells/ 185 cells). Therefore, the degree of tTA/TRE enhancement could be underestimated in above quantification.

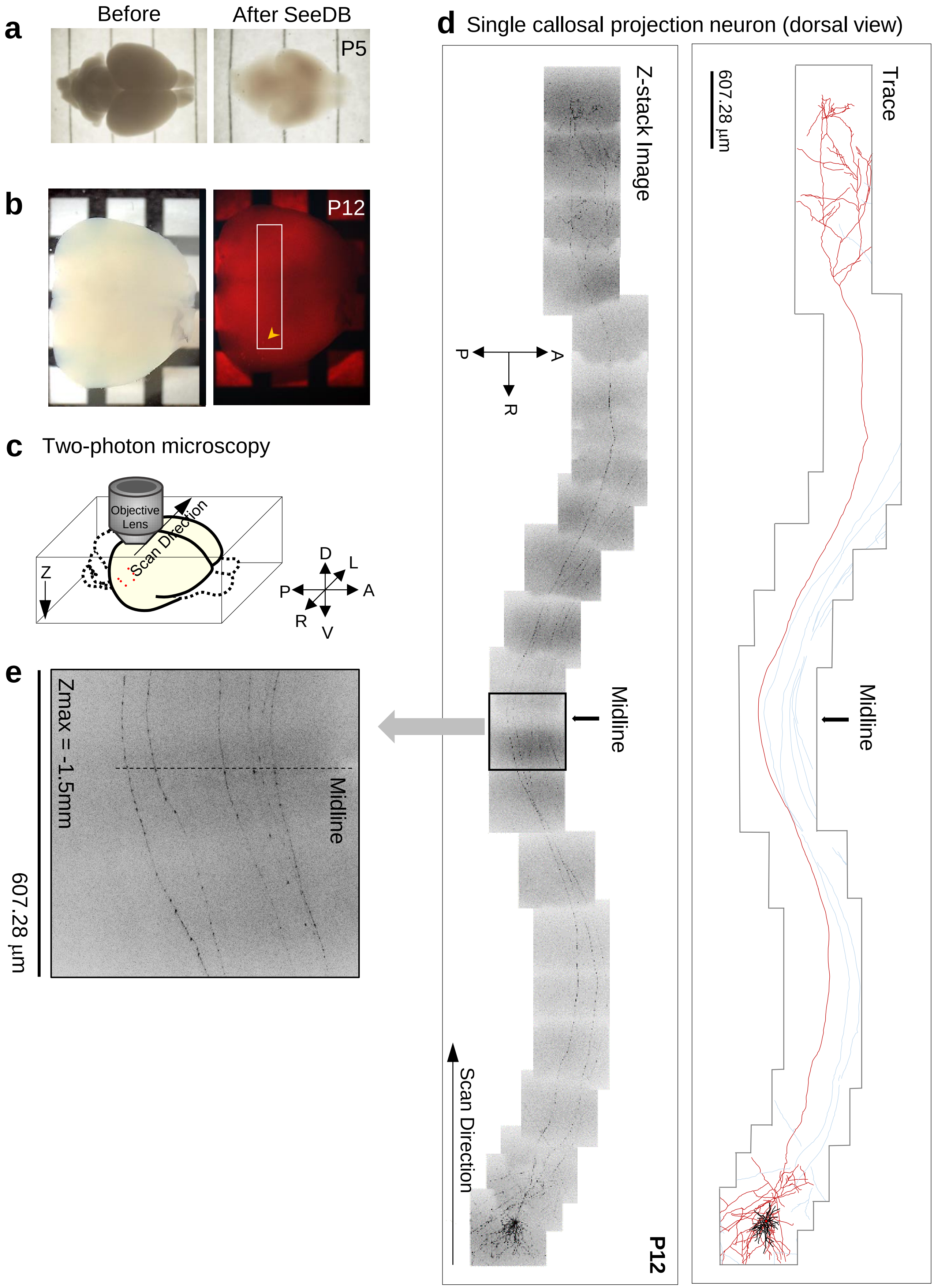
a
Before
After SeeDB
P5
b
P12
c
Two-photon microscopy
Objective Lens
Scan Direction
Z
D
L
P
A
R
V
d
Single callosal projection neuron (dorsal view)
Z-stack Image
Trace
607.28 μm
P
A
R
Midline
Midline
Scan Direction
P12
e
Zmax = -1.5mm
Midline
607.28 μm

Supplementary Figure 4. Imaging and reconstruction of an Flpe-SnRFP-labeled callosal projection neuron in intact whole brain.

(**a**) Optical clearing of the P5 mouse brain using the SeeDB method[33].

(**b-e**) Fluorescence imaging and reconstruction of an individual callosal projection neuron. (**b**) SeeDB-treated P12 brain that was electroprated with Flpe-SnRFP at E15.5. Box indicates the area imaged. (**c**) Two-photon microscopy was utilized to capture the images of a labeled neuron (arrowhead in **b** right panel). D: dorsal; V: ventral; P: posterior; A: anterior; L: left; R: Right. (**d**) Z-stacked two-photon images (left panel) and trace (right panel) of the Flpe-SnRFP-labeled callosal projection neuron. Fine morphological architecture and fluorescence signal were preserved in the SeeDB-cleared brain. Axons and dendrites of a single neuron are shown in red and black, respectively. Gray lines are axons from other neurons. All axons in the confocal image were traced. (**e**) Enlarged image of the box shown in **d** left panel. High intensity of Supernova labeling enabled clear visualization of callosal projection axons that located at 1.5mm from the cortical surface.

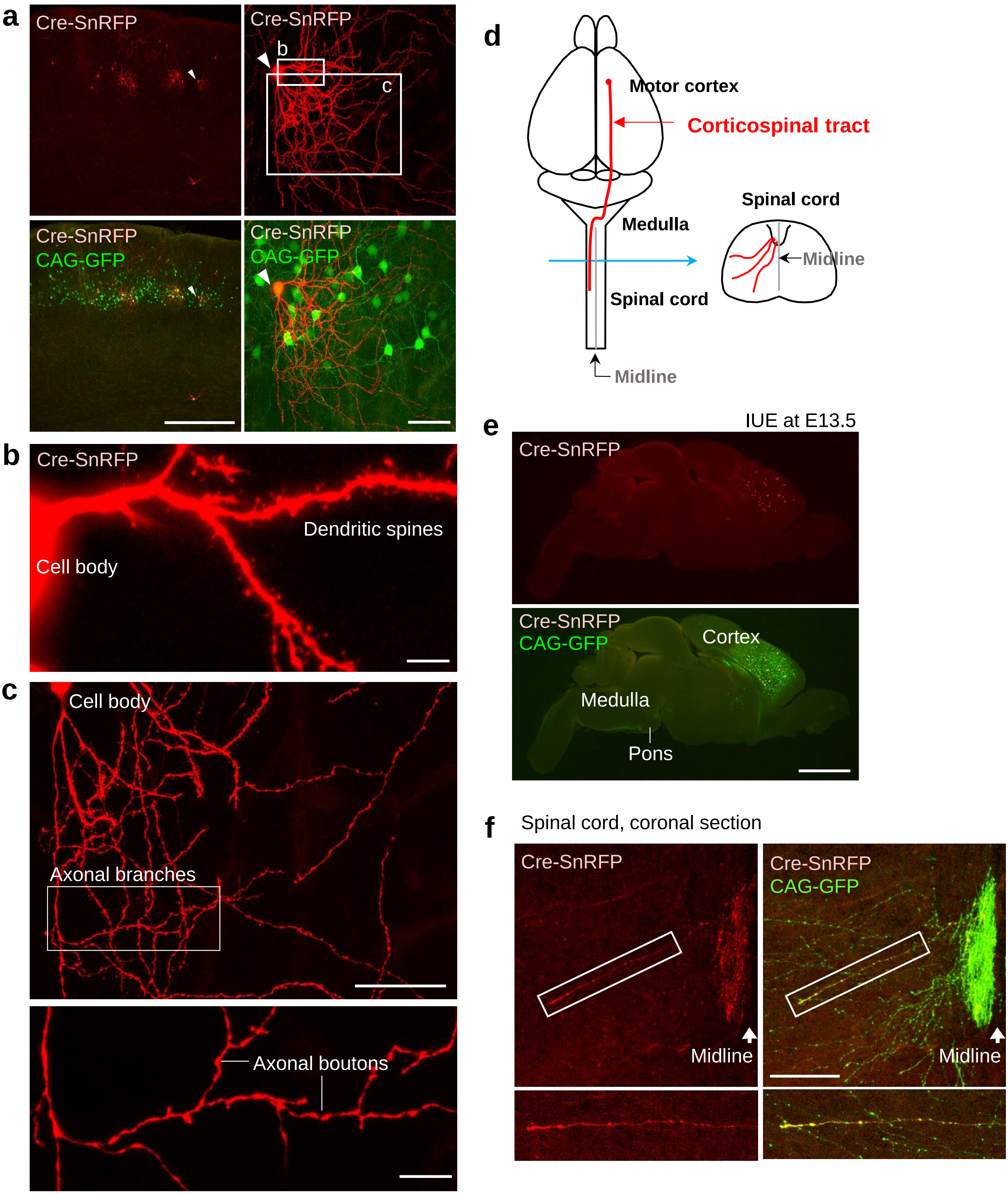

a
Cre-SnRFP
Cre-SnRFP
b
c
Cre-SnRFP
CAG-GFP
Cre-SnRFP
CAG-GFP
b
Cre-SnRFP
Dendritic spines
Cell body
c
Cell body
Axonal branches
Axonal boutons
d
Motor cortex
Corticospinal tract
Medulla
Spinal cord
Midline
Spinal cord
Midline
e
IUE at E13.5
Cre-SnRFP
Cre-SnRFP
CAG-GFP
Cortex
Medulla
Pons
f
Spinal cord, coronal section
Cre-SnRFP
Midline
Cre-SnRFP
CAG-GFP
Midline

Supplementary Figure 5. Supernova-labeling based on Cre/loxP recombination system

(**a**) Cre-based Supernova RFP (Cre-SnRFP) enabled sparse and bright neural labeling, confirming our previous results. Cre-SnRFP was electroporated into L4 cortical neurons. CAG-GFP was co-transfected to indicate the transfected neurons. Brains were cut coronally. Arrowhead indicates a Cre-SnRFP-labeled spiny stellate neuron in L4 of the somatosensory cortex. Higher-magnification images are shown in right. Scale bars: 500 μm (left panel); 50 μm (right panel).

(**b,c**) Cre-based Supernova labeling was sparse and bright enough to visualize the detailed cellular morphology, including dendritic spines (**b**), axonal branches and buttons (**c**). **c** bottom panel: a higher-magnification image of the square in the upper panel with less image stacked. Scale bars: 5 μm (**b**); 50 μm (**c**, upper panel); 7.5 μm (**c**, bottom panel).

(**d**) Schematic representation of the corticospinal tract axon (red line). Because the corticospinal tract is the longest axonal trajectory in the mammalian central nervous system, we chose it to assess the ability of Supernova in labeling long-range projection. Right panel: the coronal view of spinal cord.

(**e**) Cre-SnRFP and CAG-GFP were co-transfected into cortical neurons in the motor cortex by IUE at E13.5. Sagittal sections (100μm-thick) were made from the P5 mouse brain. Scale bar: 2.5 mm.

(**f**) Coronal sections at cervical enlargement of the spinal cord showed Cre-SnRFP-labeled single corticospinal axons. Lower panels: higher magnification images of the rectangles in the upper panels. Scale bar, 100 μm.

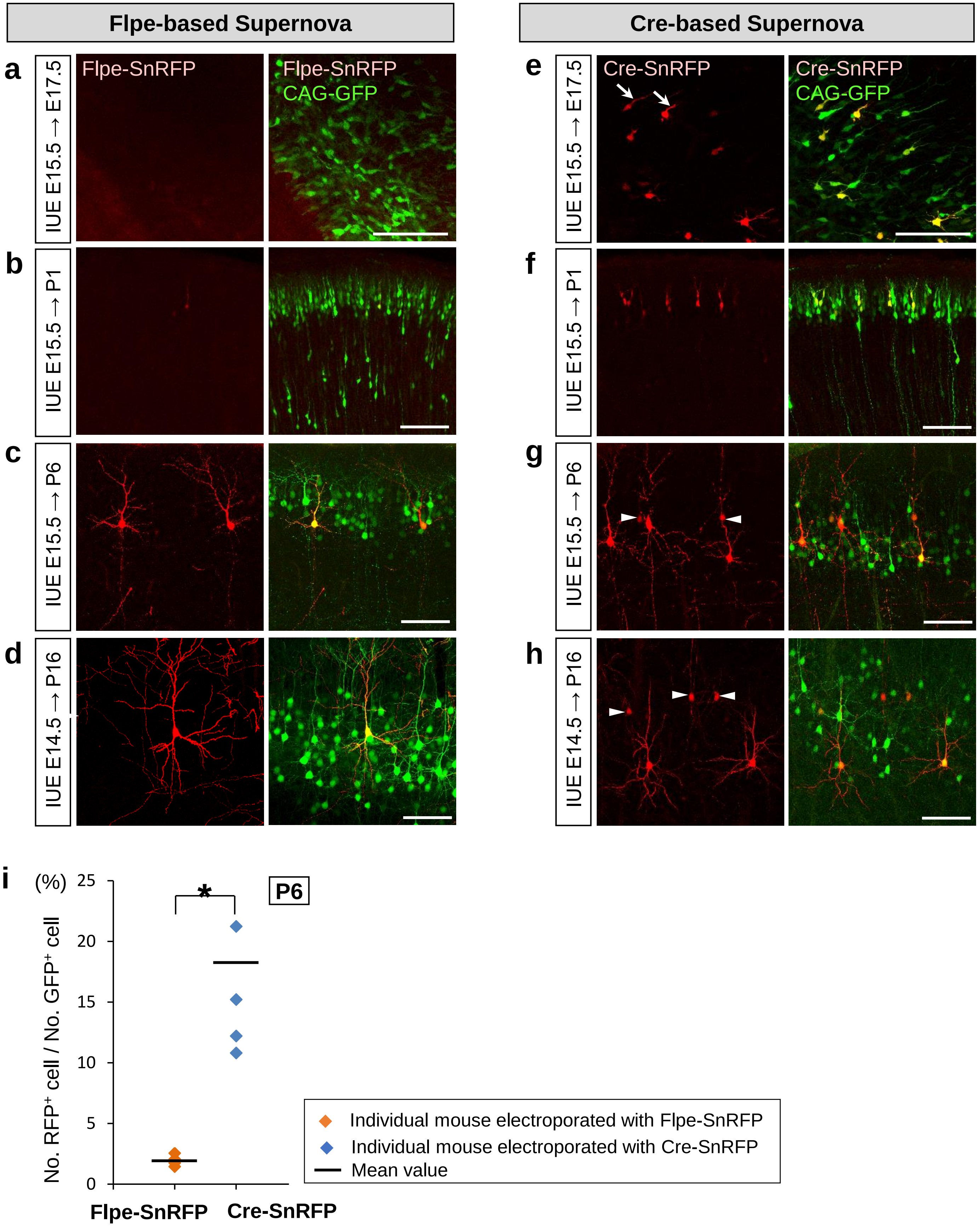
Flpe-based Supernova
Cre-based Supernova
a
b
c
d
e
f
g
h
IUE E15.5 → E17.5
IUE E15.5 → P1
IUE E15.5 → P6
IUE E14.5 → P16
Flpe-SnRFP
Flpe-SnRFP
CAG-GFP
Cre-SnRFP
Cre-SnRFP
CAG-GFP
i
(%)
P6
*
No. RFP+ cell / No. GFP+ cell
0
5
10
15
20
25
Flpe-SnRFP
Cre-SnRFP
Individual mouse electroporated with Flpe-SnRFP
Individual mouse electroporated with Cre-SnRFP
Mean value

Supplementary Figure 6. Flpe-based and Cre-based Supernova systems show some distinct features.

(**a-h**) Flpe-SnRFP (**a-d**) and Cre-SnRFP (**e-h**) were introduced into cortical neurons, separately. The CAG-GFP vector was co-transfected to indicate transfected cells. The coronal sections were made from the E17.5, P1, P6 and P16 mouse brains. The Flpe-SnRFP signal was visible 5 days after IUE (at P1) (**b**). On the other hand, Cre-SnRFP signal was observed within 2 days after transfection (at E17.5), and leading processes of labeled neurons were clearly visible (arrows) (**e**). At P1, Cre-SnRFP was already bright enough to visualize dendritic morphology of labeled neurons (**f**). At P6, the brightness was similar between Flpe-SnRFP-labeling and Cre-SnRFP-labeling (**c**,**g**). The whole cellular morphology of both Flpe-SnRFP-labeled and Cre-SnRFP-labeled neurons were clearly visualized at this age. Note that although some dark cells (48/86 cells, 4 mice at P6; arrowheads in **g**,**h**) were found in Cre-based Supernova labeling, dark cells were rarely detectable (2/28 cells, 4 mice at P6) in Flpe-RFP-labeled cortex. Scale bars: 100 μm.

(i) Compared to Cre-SnRFP, Flpe-SnRFP achieved better sparseness in neural labeling when the same concentration (5ng/μl) of TRE-SSR vector was used. At P6, 14.8%±3.3% (n=4 mice) of GFP-positive neurons were labeled by Cre-SnRFP. This ratio was significantly lower when Flpe-SnRFP was used for labeling, which was 1.9%±0.2% (n=4 mice). All values represent as mean±SEM. (*) P<0.05, Welch's *t*-test.

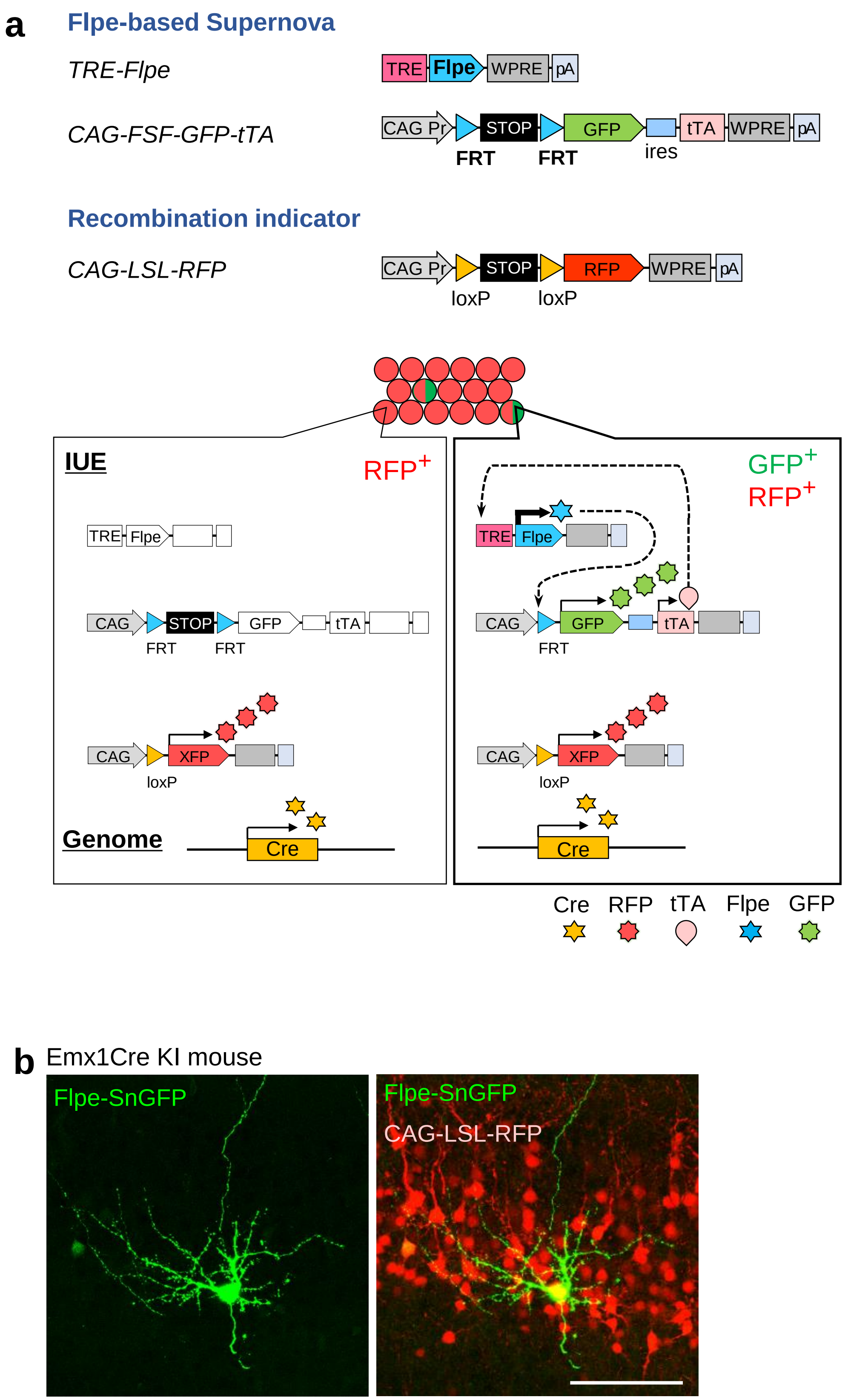
a
Flpe-based Supernova
TRE-Flpe
TRE
Flpe
WPRE
pA
CAG-FSF-GFP-tTA
CAG Pr
STOP
GFP
tTA
WPRE
pA
FRT
FRT
ires
Recombination indicator
CAG-LSL-RFP
CAG Pr
STOP
RFP
WPRE
pA
loxP
loxP
IUE
RFP+
TRE
Flpe
CAG
STOP
GFP
tTA
FRT
FRT
CAG
XFP
loxP
Genome
Cre
GFP+
RFP+
TRE
Flpe
CAG
GFP
tTA
FRT
CAG
XFP
loxP
Cre
Cre
RFP
tTA
Flpe
GFP
b
Emx1Cre KI mouse
Flpe-SnGFP
Flpe-SnGFP
CAG-LSL-RFP

Supplementary Figure 7. Supernova systems based on Flpe/FRT and Dre/rox enabled sparse neuronal labeling in Cre-expressing mice.

(**a**) Illustrations show what happens in neurons transfected by Flpe-SnGFP (TRE-Flpe and CAG-FSF-GFP-tTA) and CAG-loxP-stop-loxP-RFP-WPRE-pA (CAG-LSL-RFP) in Cre-expressing areas. (**b**) When the CAG-LSL-RFP vector was introduced into the cortex of Emx1Cre knock-in (KI) mice, which exhibit Cre-mediated recombination in all excitatory neurons in the cortex[32,38], neurons were labeled densely. In contrast, the Flpe-SnGFP vector set introduced together with the CAG-LSL-RFP vector labeled only a small population of cortical neurons, and dendritic morphologies of these SnGFP-labeled neurons were clearly visible. All Supernova-positive cells co-localized with Cre-expressing cells. IUE were performed at E14.5, the brains were dissected at P8 and coronal sections were made. Scale bar in **b**, 100 μm.

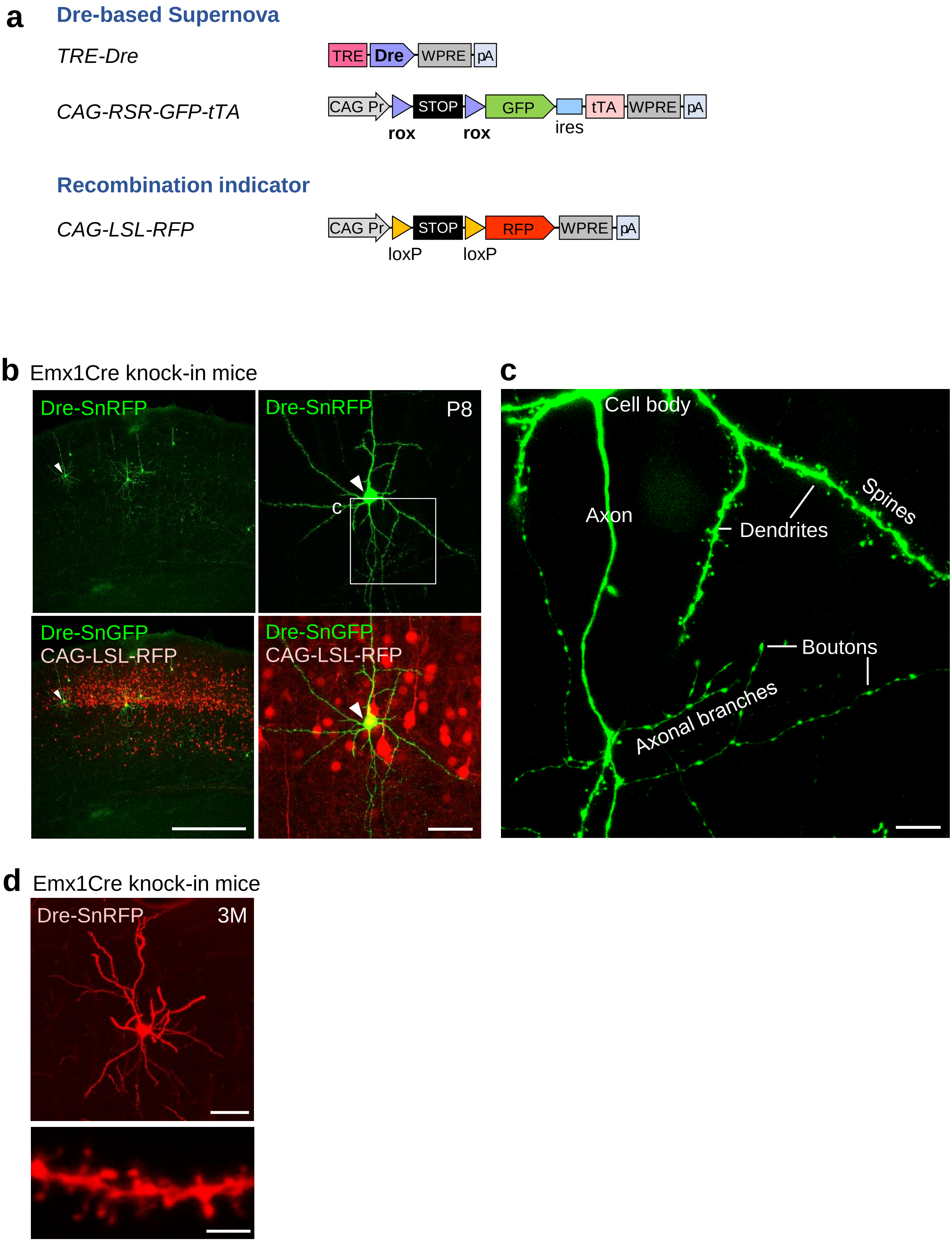
a
Dre-based Supernova
TRE-Dre
TRE
Dre
WPRE
pA
CAG-RSR-GFP-tTA
CAG Pr
STOP
GFP
tTA
WPRE
pA
rox
rox
ires
Recombination indicator
CAG-LSL-RFP
CAG Pr
STOP
RFP
WPRE
pA
loxP
loxP
b
Emx1Cre knock-in mice
Dre-SnRFP
Dre-SnRFP
P8
c
Dre-SnGFP
CAG-LSL-RFP
Dre-SnGFP
CAG-LSL-RFP
c
Cell body
Axon
Dendrites
Spines
Boutons
Axonal branches
d
Emx1Cre knock-in mice
Dre-SnRFP
3M

Supplementary Figure 8. Supernova labeling based on Dre/rox recombination system.

(**a**) Schematics showing Dre-based Supernova GFP (Dre-SnGFP) vector set (TRE-Dre and CAG-rox-stop-rox-GFP-tTA) and Cre-mediated recombination indicator (CAG-LSL-RFP).

(**b**) Supernova vector set based on Dre/rox recombination also enabled sparse and bright neural labeling. The whole cellular morphology can be clearly seen. Dre-based Supernova GFP (Dre-SnGFP) vector set (TRE-Dre and CAG-rox-stop-rox-GFP-tTA) was introduced into L4 cortical neurons of Emx1Cre knock-in (KI) mice[38] by IUE at E14.5. CAG-LSL-RFP vector was co-transfected. Right panel shows higher-magnification images of Dre-SnGFP labeled cortical neuron (arrows in left panels). Scale bars: 500 μm (left panel); 50 μm (right panel).

(**c**) The sparse and bright labeling via Dre-SnGFP enabled clear visualization of dendritic spines, axonal branches and axonal buttons of labeled neurons in **b** right panel. Scale bar, 10 μm.

(**d**) The high intensity of Dre-based Supernova RFP (Dre-SnRFP)-labeling was maintained even in adult (3-month-old, 3M) mice, and enabled clear visualization of whole cellular morphology including dendritic spines. Scale bars: 50 μm (upper panel); 2.5 μm (bottom panel).

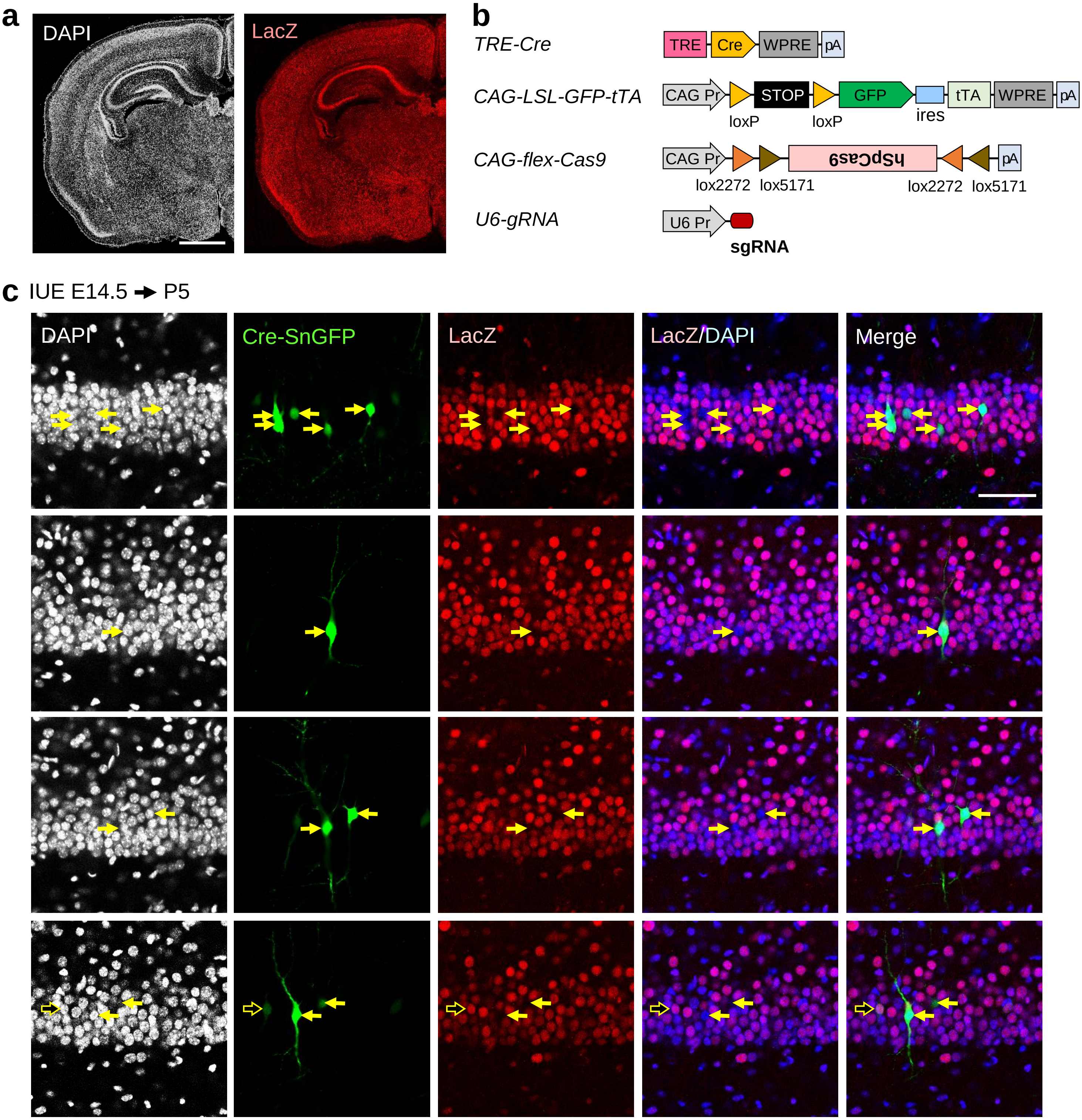
a
DAPI
LacZ
b
TRE-Cre
TRE
Cre
WPRE
pA
CAG-LSL-GFP-tTA
CAG Pr
STOP
GFP
tTA
WPRE
pA
loxP
loxP
ires
CAG-flex-Cas9
CAG Pr
hSpCas9
pA
lox2272
lox5171
lox2272
lox5171
U6-gRNA
U6 Pr
sgRNA
c
IUE E14.5 → P5
DAPI
Cre-SnGFP
LacZ
LacZ/DAPI
Merge

Supplementary Figure 9. Supernova-mediated CRISPR/Cas9 effectively disrupted target gene expression at a single-cell level in vivo.

(**a**) Rosa-LacZ mice ubiquitously express LacZ. Coronal sections (60μm-thick) were made from the P5 Rosa-LacZ mouse brain and stained with an anti-β-gal antibody. Scale bar, 1 mm.

(**b**) Schematic representation of a Supernova-CRISPR/Cas9 vector set for single-cell genome editing. CAG-flex-Cas9 and U6-gRNA targeting LacZ vectors were described elsewhere[12].

(**c**) LacZ expression was undetectable in most SnGFP-labeled hippocampal neurons (arrows, 39/45cells, 3 mice), although LacZ was ubiquitously expressed in surrounding GFP-negative neurons. Only in a few cases, LacZ expression was still detectable (hollow arrows, 6/45cells, 3 mice) in SnGFP-labeled neurons. Supernova-CRISPR/Cas9 that targets LacZ was electroporated into the hippocampus of Rosa-LacZ mice. LacZ expression was detected by an anti-β-gal antibody. DAPI staining was performed to visualize the cell-body localization. Scale bar, 50 μm.

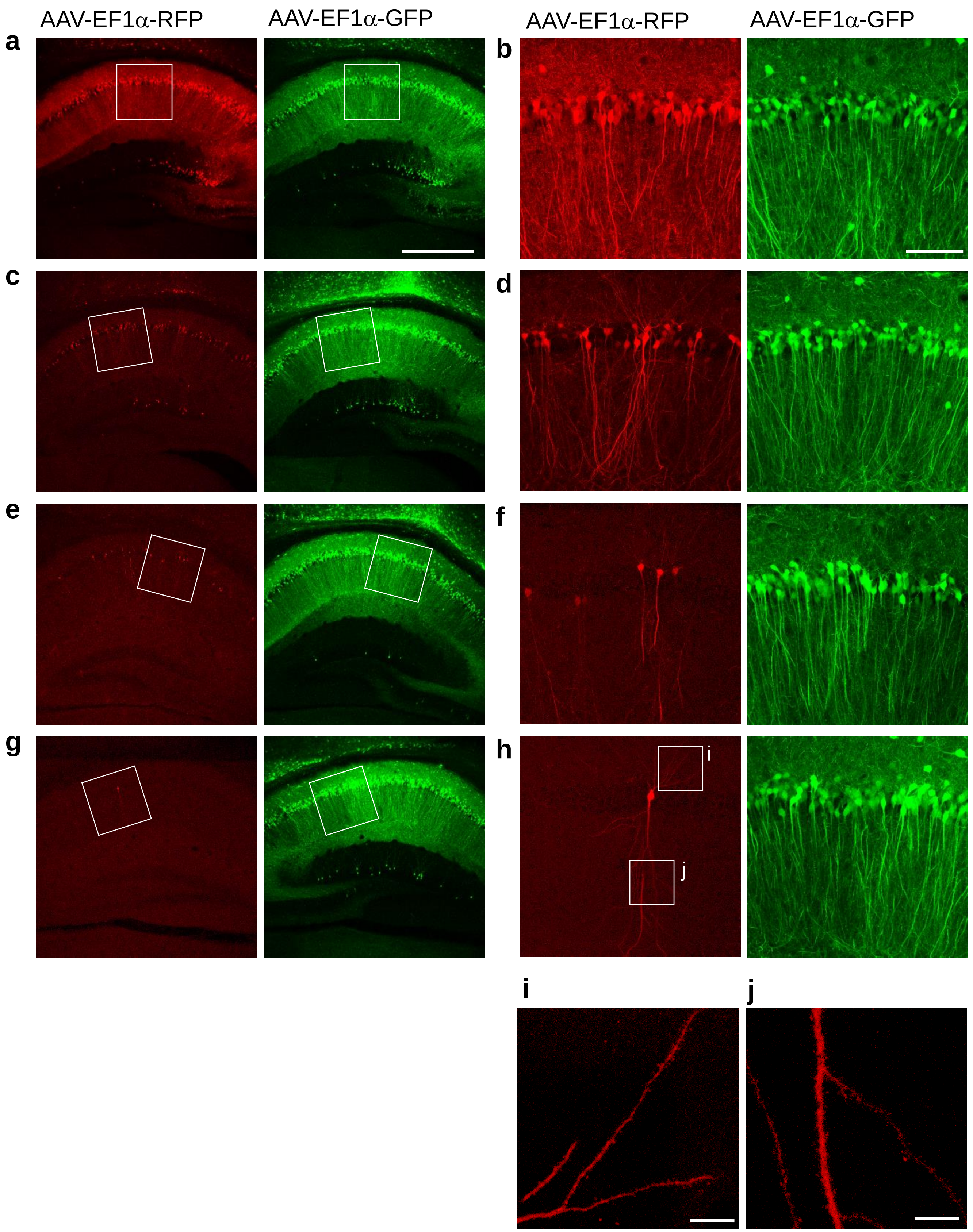
AAV-EF1α-RFP
AAV-EF1α-GFP
AAV-EF1α-RFP
AAV-EF1α-GFP
a
b
c
d
e
f
g
h
i
j
i
j

Supplementary Figure 10. Dilution of conventional AAV vector resulted in sparse but dark cell labeling.

(**a-h**) Hippocampal CA1 neurons labeled by AAV-EF1α-RFP-WPRE. The titer of original AAV stock was 5.5×10^{11} genome copies/ml. Non-diluted virus or virus diluted 10-10^3 folds in PBS was injected into P10 mouse brains (a-b: non-dilution, c-d:10-fold dilution, e-f:10^2-fold dilution, g-h:10^3-fold dilution; n=3 mice for each group). Infected brains were sampled at 36DPI. AAV-EF1α-GFP-WPRE was co-injected as control. (**a,c,e,g**): low-power images. Scale bar, 500 μm. (**b,d,f,h**): higher-magnification images of squares in **a,c,e,g**. Scale bar, 100 μm.

(**i,j**) Higher-magnification images of the squares in **h**, showing the basal (**i**) and apical (**j**) dendrites of sparsely labeled CA1 neuron. In **i** and **j**, less images were stacked. The labeling is extremely faint that the dendritic branches and spines are hardly seen. Scale bars, 10 μm.

AAV-SnRFP labeled cortical neuron, 10DPI

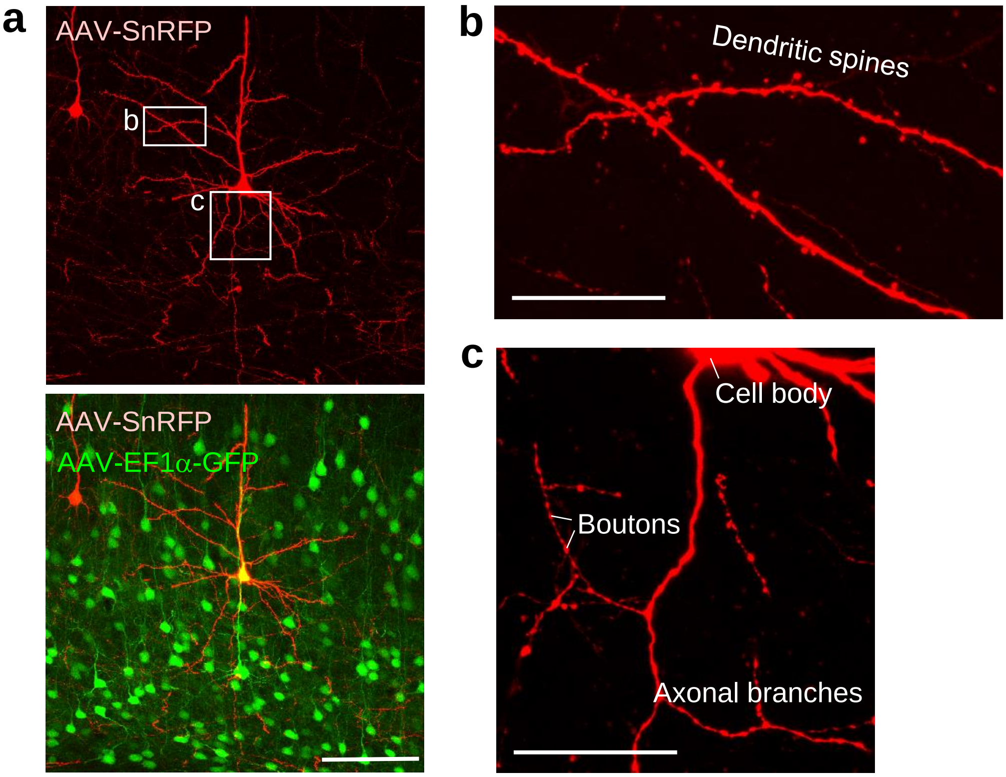

AAV-SnRFP labeled hippocampal CA1 neuron, 10DPI

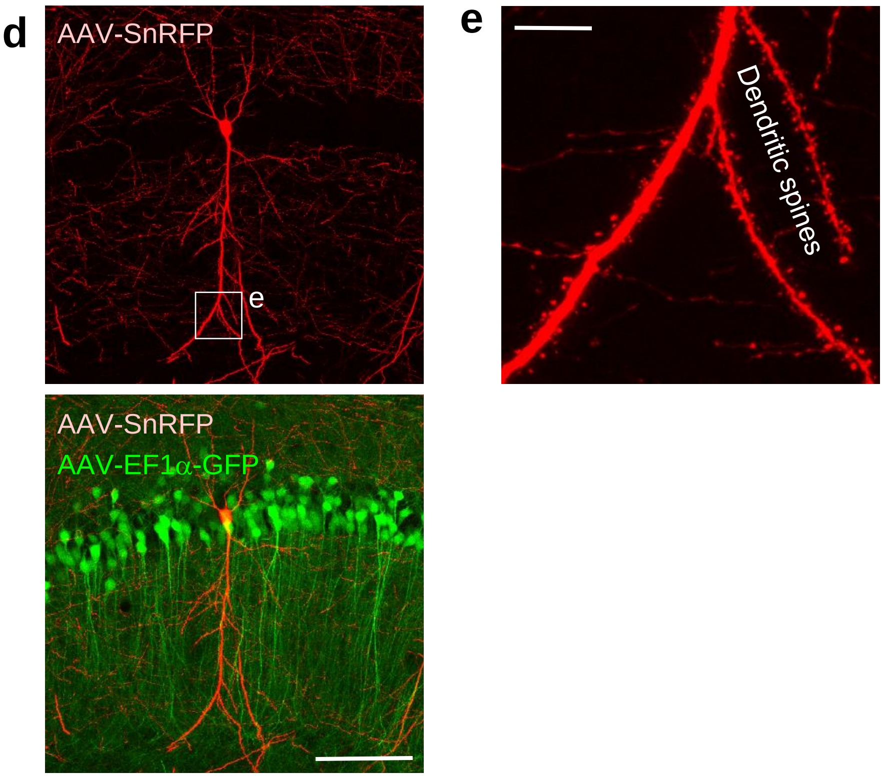

Supplementary Figure 11. Dendritic spines of AAV-SnRFP-labeled neurons can be clearly observed at 10DPI.

The detailed cellular morphology of AAV-SnRFP-labeled cortical neuron (**a-c**) and hippocampal neuron (**d,e**) can already been clearly visualized at 10 days after transfection. AAV-SnRFP was injected to P12 mouse brain. Brains were sampled at 10DPI and coronal sections were prepared. **b,c** and **e** are higher-magnification images of the rectangles in **a** and **d**. Scale bars, 100μm (**a,d**), 25μm (**b,c**), 10μm (**e**).

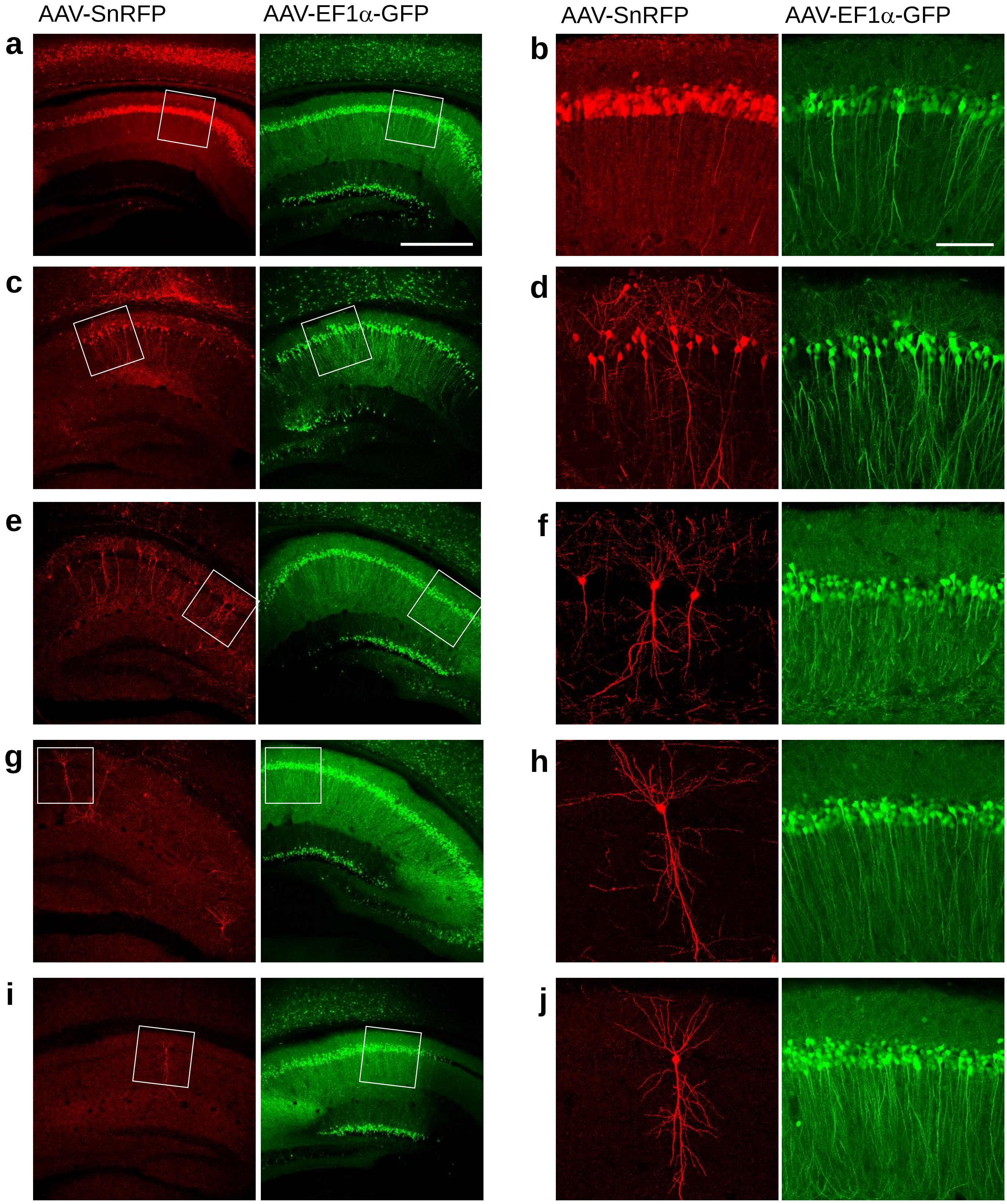

AAV-SnRFP
AAV-EF1α-GFP
AAV-SnRFP
AAV-EF1α-GFP
a
b
c
d
e
f
g
h
i
j

Supplementary Figure 12. Labeling sparseness is adjustable in AAV-Supernova without affecting labeling brightness.

Sparseness of AAV-SnRFP labeling was adjustable by simply changing the concentration of the AAV-TRE-Cre vector in viral mixture. Not like conventional AAV labeling (**Supplementary Fig. 10**), the labeling brightness was not changed by altering labeling sparseness. For AAV-SnRFP labeling, AAV-TRE-Cre-WPRE (2.3×10^{13} genome copies/ml) was diluted 10^2-10^6 folds in PBS. Equal amount of diluted AAV-TRE-Cre-WPRE and AAV-EF1α-DIO-tTA-RFP (1.7×10^{13} genome copies/ml) was mixed and injected into P11-P13 mouse brains (Dilution of AAV-TRE-Cre; **a-b**, 10^2-fold dilution; **c-d**:10^3-fold dilution; **e-f**:10^4-fold dilution; **g-h**:10^5-fold dilution; **i-j**:10^6-fold dilution. n=3 mice for each group). AAV-EF1a-GFP-WPRE (3.5×10^{11} genome copies/ml) co-infected as control. Brains were fixed at 30DPI. (**a,c,e,g,i**): low-power images. Scale bar, 500 μm. (**b,d,f,h,j**): higher-magnification images of squares in **a,c,e,g,i**. Scale bar, 100 μm.

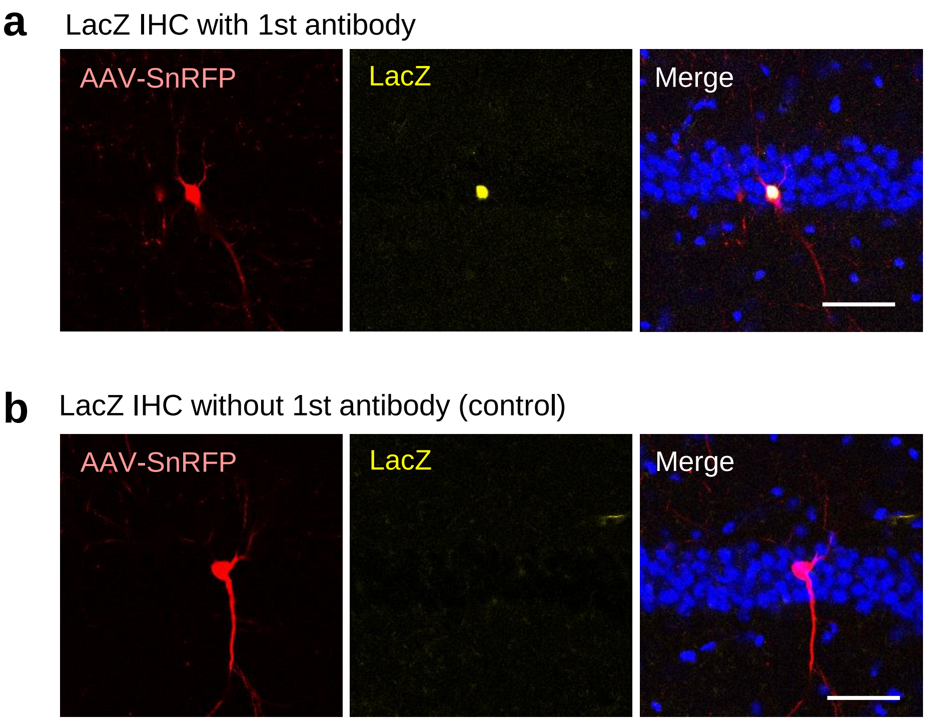
a
LacZ IHC with 1st antibody
AAV-SnRFP
LacZ
Merge
b
LacZ IHC without 1st antibody (control)
AAV-SnRFP
LacZ
Merge

Supplementary Figure 13. LacZ expression is detected specifically in Supernova-labeled neurons in RNZ reporter mice

(**a,b**) Related to **Fig.8h**. Cre-mediated genomic recombination in Rosa26-loxP-stop-loxP-nlsLacZ (RNZ) reporter mouse was detected only in SnRFP-labeled neurons in hippocampus CA1 (**a**). Observed LacZ signals were not derived from the leakage of the strong AAV-SnRFP signal, because no LacZ signal was detected if the 1st antibody was not added (**b**). Scale bar, 50μm.

Supplementary Table 1. Vectors used in this study.

Vector #	Vector Name
pK013	pBS302(loxP-stop-loxP)
pK016	pCAG-Flpe-ires-Puro (GeneBridge)
pK021	pLenti-Synapsin-hChR2 (H134R)-EYFP-WPRE
pK023	pTurboRFP-N (Evrogen)
pK024	pCAsalEGFP
pK025	pCAG-TurboRFP
pK026	pTRE-Tight vector (Clontech)
pK029	pCAG-loxP-STOP-loxP-RFP-ires-tTA-WPRE
pK031	pTRE-Cre
pK036	pTRE-Flpe-WPRE
pK037	pCAG-FRT-STOP-FRT-RFP-ires-tTA-WPRE
pK038	pCAG-loxP-STOP-loxP-EGFP-ires-tTA-WPRE
pK039	pCAG-loxP-STOP-loxP-AmCyan-ires-tTA-WPRE
pK046	pCAG-loxP-STOP-loxP-RFP-WPRE
pK055	pCAG-Dre-ires-puro.gcc vector
pK068	pCAG-FRT-STOP-FRT-EGFP-ires-tTA-WPRE
pK073	pTRE-Dre-WPRE
pK078	pJFRC176-10XUAS-rox-dSTOP-rox-myr::GFP (Addgene)
pK098	pCAG-loxP-STOP-loxP-nlstagRFP-ires-tTA-WPRE
pK102	pCAG-loxP-STOP-loxP-PSD95eGFP-ires-tTA-WPRE
pK129	pCAG-Rox-STOP-Rox-RFP-ires-tTA-WPRE
pK162	pX330
pK165	pAAV-EF1α-RFP-WPRE
pK168	pAAV-EF1α-DIO-RFP-P2A-tTA-WPRE
pK169	pAAV-TRE-MCS-WPRE
pK170	pAAV-TRE-Cre-WPRE
pK177	pCAG-loxP-STOP-loxP-mir30 (GFP RNAi)
pK178	pCAG-loxP-STOP-loxP-mir30 (GFP RNAi Scramble control)
pK209	pCMV-α2-Chn-TALEN Left
pK210	pCMV-α2-Chn-TALEN Right
pK217	pCAG-FRT-STOP-FRT-α2Chn TALEN Left
pK218	pCAG-FRT-STOP-FRT-α2Chn TALEN Right
pK224	pCAG-Rox-STOP-Rox-EGFP-ires-tTA-WPRE
pK225	pCAG-loxP-STOP-loxP-mir30 (LacZ RNAi)
pK226	pCAG-loxP-STOP-loxP-mir30 (LacZ RNAi Scramble control)
pK229	pAAV-EF1α-EGFP-WPRE

pK232	pCAG-flex-3Flag-hspCas9
pK233	pX330-U6-gRNALacZ
pK237	pU6-gRNA-CAG-LSL-hspCas9 (add LSL to pX330)
pK238	pU6-gRNACreb1(Wl107/108)-CAG-LSL-hspCas9

Supplementary Table 2. Primers used in this study.

Primer	Primer Sequence (from 5' to 3')
HM050	CGGTCGACGCCACCATGGTGAGCAAGGGCGAGGAGC
HM055	GGAATCCTTAATTAACTCGATCTAGGATATCTTACTTGTACAGCTCGTCCATGCCGAGA
HM054	TCTCGGCATGGACGAGCTGTACAAGTAAGATATCCTAGATCGAGTTAATTAAGGATTCC
HM035	GGGCGGCCGCTTACTTAGTTACCCGGGGAGCATGTCAAG
HM079	CCGTCGACCATGGGCCCAAAGAAGAAGAGAAAGGTTTCGGTGTCTAAGGGCGAAG
HM075	CCGATATCTTCAATTAAGTTGTGCCCCAGTTTGCTAGG
AY101	GGAAGGATCCTTGTGCTGTCTCATCATTTTGG
AY102	TTGCGGCCGCGGGCGGAATTCTTAATTAACTC
HM008	GGGAATTCTAGAAGTTCCTATACTTTCTAGAGAATAGGAACTTCATTAAGGGTTCCGGATCCTCGGGGACACCA
HM009	CCGTCGACATGAAGTTCCTATTCTCTAGAAAGTATAGGAACTTCTAGGTCCCTCGACCTGCAGCCCAAGCTTA
WL006	CGAATTCGCC ACCATGGGTG CT
WL007	TGCGGCCGCT CACACTTTCC TCTT
HM084	TGCTGTTGACAGTGAGCGAAGCCACAACGTCTATATCATGTAGTGAAGCCACAGATGTACATGATATAGACGTTGTGGCTGTGCCTACTGCCTCGGA
HM085	TGCTGTTGACAGTGAGCGAGCTCATAGAAGCTCTACAATCTAGTGAAGCCACAGATGTAGATTGTAGAGCTTCTATGAGCGTGCCTACTGCCTCGGA
HM082	CAGAAGGCTCGAGAAGGTATATTGCTGTTGACAGTGAGCG
HM083	CTAAAGTAGCCCCTTGAATTCCGAGGCAGTAGGCA
HM088	GCGGCCGCATCGATGATATC
HM089	GATAGGCAGCCTGCACCTGAG
WL090	TGCTGTTGACAGTGAGCGAAAATCGCTGATTTGTGTAGTCTAGTGAAGCCACAGATGTAGACTACACAAATCAGCGATTTGTGCCTACTGCCTCGGA
WL091	TGCTGTTGACAGTGAGCGAGGCAACTTCGGTTAGTATTTATAGTGAAGCCACAGATGTATAAATACTAACCGAAGTTGCCGTGCCTACTGCCTCGGA
WL107	CACCGTTGTCTGCTCCAGATTCCA
WL108	AAACTGGAATCTGGAGCAGACAAC
HM010	GGGAATTCTAATAACTTCGTATAGCATACATTATACGAAGTTATATTAAGGGTTCCGGATCCTCGGGGACACC
HM007	CCGTCGACATATAACTTCGTATAATGTATGCTATACGAAGTTATTAGGTCCCTCGACCTGCAGCCCAAGCTTA
HM026	CGAATTCTGCAGTCGACGGTACCGAGGGCC
HM030	GTCGCGGCCGCATCATCTGTGCCCCAGTTT
HM019	GCGGCCGCTCAACCTCTGGATTACAAAATT
HM020	GCGGCCGCTGCGGGGAGGCGGCCCAAAGGG
WL105	GCTTTGAGTCCATCCACGAT

WL106	GCAGGGACTCCCAATAGACA
WL111	CACCACGGCCACCGATATTA
WL112	GTATCGCCAAAATCACCGCC
WL113	GCATTTGCTAACTTAACCACCACA
WL114	GGCTGGGCTTGAACTGTCAT
RY78	5’-TGCTAGCGCCACCATGTCTAGACTGGA-3’
RY79	5’-TGGATCCCCCGGGGAGCATGTCAAGGT-3’
RY69	5’-AGGATCCGGAAGCGGAGCTACTAACTTCAGCCTGCTGAAGCAGGCTGGAGACGTGGAGGAGAACCCTGGACCTATGAGCGAGCTGATCA-3’
RY80	5’-AGGCGCGCCTCATCTGTGCCCCAGTTTG-3’
WL103	5’-CTGAATTCGCCACCATGGTGAGCAAGGGCGAGGAGC-3’
WL104	5’-CGGATCCTTACTTGTACAGCTCGTCCATGCCGAGAG-3’
WL123	5’-GTATGGAGCAAGGGGCAAG-3’
WL124	5’-AGGCGGAGATTGCAGTGAG-3’

In order of appearance in the Online Methods.
